# Combining TSS-MPRA and sensitive TSS profile dissimilarity scoring to study the sequence determinants of transcription initiation

**DOI:** 10.1093/nar/gkad562

**Published:** 2023-07-05

**Authors:** Carlos Guzman, Sascha Duttke, Yixin Zhu, Camila De Arruda Saldanha, Nicholas L Downes, Christopher Benner, Sven Heinz

**Affiliations:** Department of Medicine, Division of Endocrinology, U.C. San Diego School of Medicine, La Jolla, CA 92093, USA; Department of Bioengineering, Graduate Program in Bioinformatics & Systems Biology, U.C. San Diego, La Jolla, CA 92093, USA; Department of Medicine, Division of Endocrinology, U.C. San Diego School of Medicine, La Jolla, CA 92093, USA; Department of Medicine, Division of Endocrinology, U.C. San Diego School of Medicine, La Jolla, CA 92093, USA; Department of Medicine, Division of Endocrinology, U.C. San Diego School of Medicine, La Jolla, CA 92093, USA; Department of Medicine, Division of Endocrinology, U.C. San Diego School of Medicine, La Jolla, CA 92093, USA; Department of Medicine, Division of Endocrinology, U.C. San Diego School of Medicine, La Jolla, CA 92093, USA; Department of Medicine, Division of Endocrinology, U.C. San Diego School of Medicine, La Jolla, CA 92093, USA

## Abstract

*Cis*-regulatory elements (CREs) can be classified by the shapes of their transcription start site (TSS) profiles, which are indicative of distinct regulatory mechanisms. Massively parallel reporter assays (MPRAs) are increasingly being used to study CRE regulatory mechanisms, yet the degree to which MPRAs replicate individual endogenous TSS profiles has not been determined. Here, we present a new low-input MPRA protocol (TSS-MPRA) that enables measuring TSS profiles of episomal reporters as well as after lentiviral reporter chromatinization. To sensitively compare MPRA and endogenous TSS profiles, we developed a novel dissimilarity scoring algorithm (WIP score) that outperforms the frequently used earth mover's distance on experimental data. Using TSS-MPRA and WIP scoring on 500 unique reporter inserts, we found that short (153 bp) MPRA promoter inserts replicate the endogenous TSS patterns of ∼60% of promoters. Lentiviral reporter chromatinization did not improve fidelity of TSS-MPRA initiation patterns, and increasing insert size frequently led to activation of extraneous TSS in the MPRA that are not active *in vivo*. We discuss the implications of our findings, which highlight important caveats when using MPRAs to study transcription mechanisms. Finally, we illustrate how TSS-MPRA and WIP scoring can provide novel insights into the impact of transcription factor motif mutations and genetic variants on TSS patterns and transcription levels.

## Graphical Abstract

**Graphical Abstract:**
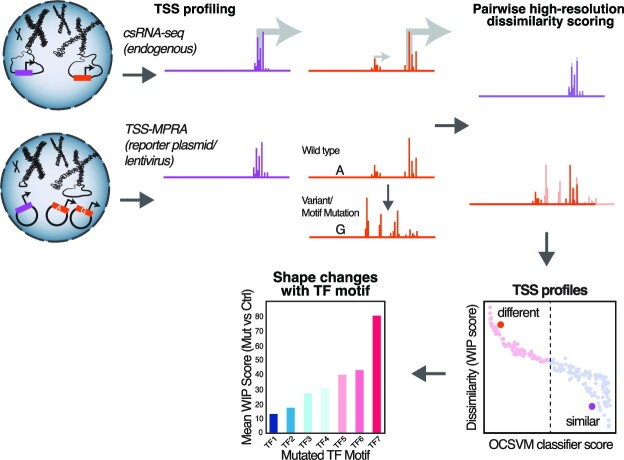

## INTRODUCTION

Recent advances in next-generation sequencing have broadened the molecular toolkit available to study the link between *cis*-regulatory grammar—the set of rules that govern the process by which DNA-encoded information is interpreted by transcription factors and other regulatory proteins—and regulatory element function. In particular, new methods capable of detecting transcriptional start sites (TSSs) such as CAGE (1), Start-seq (2), GRO-cap (3,4), csRNA-seq (5) and STAP-seq (6–8) have allowed researchers to discover and investigate previously underappreciated features of regulatory grammar. For instance, base-resolution identification of TSSs have revealed that enhancers, which are frequently transcribed (9,10), strongly resemble gene promoters at the sequence level (4).

Promoters can be classified by the shape of their transcription initiation patterns. Focused (sharp) promoters have a single, well-defined TSS, while dispersed (broad) promoters have multiple TSSs that cluster closely together in linear space (11). Whereas focused promoters tend to drive the transcription of cell type- or stimulus-specific genes, dispersed promoters are often associated with housekeeping genes (12). Focused and dispersed initiation patterns have been linked to distinct families of transcription factors and core promoter elements (11,13). Furthermore, changes in transcription factor binding near TSSs have been shown to affect TSS profile shape and nucleosome positioning at promoters and to result in aberrant gene expression (14). Similarly, studies of quantitative trait loci (QTL) impacting TSS patterns in *Drosophila melanogaster* suggest that genomic variants that affect promoter TSS profile shape often increase transcriptional noise (15). Taken together, these results provide evidence that TSS profiles reflect regulatory element function (15) and that differences between transcription initiation patterns point to differences in regulatory mechanisms (7,16–17).

Interrogating the relationship between *cis*-regulatory sequence and function *in vivo* remains laborious and low throughput, largely due to the difficulties posed by editing genomic sequences in living cells. Massively parallel reporter assays (MPRAs) overcome these limitations by enabling functional screening of thousands of sequences for regulatory activity in parallel, making them increasingly popular tools to analyze *cis-*regulatory element (CRE) function (6–8,18–33).

Broadly speaking, MPRAs either rely on RNA-seq or DNA-seq after fluorescence-activated cell sorting (FACS) to measure transcriptional output of reporter constructs. RNA-seq-based MPRA approaches quantify CRE activity by counting unique CRE-associated sequences encoded in the reporter RNA transcript (either synthetic barcodes (18) or the transcribed CRE sequence itself (23)). FACS-based Sort-seq approaches (19) measure CRE activity by FACS-sorting cells into bins based on reporter gene expression and counting relative plasmid CRE abundance in each bin after high-throughput sequencing. RNA-seq MPRAs are more prevalent than Sort-seq MPRAs, but Sort-seq MPRAs have played and continue to play important roles in the quantitative study of transcriptional regulation (e.g. (22,30)).

One benefit of RNA-seq-based MPRAs is that they can be modified to provide data on reporter transcript initiation patterns (6,25–26,33), which are indicative of regulatory mechanisms (7,17,34). This opens the door for using transcription initiation profiles to study the grammar of genomic promoter sequences and to distinguish the effects of human genetic variants on transcription initiation from the effects on transcription levels. Therefore, it is essential to know how well MPRAs recapitulate endogenous initiation profiles when using them to study regulatory mechanisms. The only prior study that touched on this subject has suggested that TSS patterns in an MPRA match endogenous TSS patterns, based on the analysis of averaged initiation signals across thousands of *Drosophila* promoters (6). However, it is well known that promoters exhibit a wide variety of TSS patterns (11), and no study to date has performed a detailed analysis of individual promoters. Hence, the degree to which individual MPRA constructs recapitulate the transcription initiation patterns of the corresponding endogenous promoters still remains unexplored.

Here, we present the first analysis of TSS pattern fidelity of individual promoters in an MPRA compared to the genome. In the process of analyzing our data we found that previously used computational approaches to compare TSS patterns did not adequately quantify the magnitude of the observed TSS pattern differences. In an effort to better capture the degree to which MPRA inserts replicate the transcription initiation landscape of endogenous sequences, we developed a windowed initiation profile (WIP) score that takes full advantage of the single-nucleotide resolution transcription initiation data generated by 5′ RNA sequencing methods. Using it to compare TSS-MPRA-derived and endogenous initiation patterns of individual human promoters as determined by csRNA-seq, we find that MPRA initiation profiles of short 153-bp promoter inserts correctly replicate ∼60% of the endogenous initiation patterns of the human promoters we tested. Unexpectedly, for the remaining promoters, neither increasing the insert size nor lentiviral reporter chromatinization improved the fidelity of their MPRA-derived transcription initiation profiles. Instead, longer inserts frequently gave rise to additional, extraneous initiation events, decreasing overall TSS profile fidelity when comparing TSS-MPRA to endogenous initiation profiles. We discuss the putative causes for this observation and demonstrate how TSS-MPRA and TSS profile scoring can be used to study the transcriptional effects of human genetic variants. We anticipate that our new low-input protocol, the lentiviral TSS-MPRA vector and novel sensitive TSS profile analysis approach will broaden the utility of TSS-aware MPRAs for studying the effect of human genetic variation. In addition, our results highlight important caveats to consider when using MPRAs to decipher DNA transcription regulatory function.

## MATERIALS AND METHODS

### Experimental methods

#### TSS-MPRA plasmid designs

The episomal TSS-MPRA plasmid is based on the background-reduced pGL4.10 luciferase reporter plasmid (Promega). To allow directional cloning of oligonucleotide libraries via Gibson assembly, we replaced the multiple cloning site and Luciferase 2 gene of pGL4.10 with a new eGFP reporter cassette to quantify insert-initiated transcription by fluorescence microscopy and flow cytometry and an upstream cloning site containing dual *BsaI* restriction sites. The latter are flanked by an arbitrary upstream 18-nt sequence for Gibson assembly (5′-GGTAACCGGTCCAGCTCA-3′) and downstream binding sites for Illumina TruSeq Read 2 (5′-AGACGTGTGCTCTTCCGATCT-3′) and RS2 reverse transcription (RT) primers (5′-AGCGGATAACAATTTCACACAGGA-3′) for reporter-specific reverse transcription and generation of 5′-RNA-seq libraries, respectively. The resulting pTSS-MPRA-Empty plasmid was sequence-verified by Sanger sequencing.

The pLenti-TSS-MPRA plasmid for the lentiviral integration experiments was generated by replacing the multiple cloning site of pLS-SceI (35) between the *SbfI* and *AgeI* sites with a cassette containing dual *BsmBI* restriction sites and flanking upstream cloning and downstream TruSeq Read 2 and RS2 RT primer landing sites described above by Gibson assembly. The resulting pLenti-TSS-MPRA-Empty plasmid was sequence-verified by Sanger sequencing.

#### 200-bp insert library cloning

Ten nanograms (see also Supplementary Note 1) of a 200-nt DNA oligonucleotide pool containing 2000 insert/barcode combinations (Twist Biosciences) (Supplementary Table S1) were PCR-amplified in a 50 μl volume [25 μl NEBNext Ultra II Q5 Master Mix (NEB), 0.25 μl 100 μM pMPRA1-LH (5′-GGTAACCGGTCCAGCTCA-3′), 0.25 μl 100 μM pMPRA1-RH (5′-CGTGTGCTCTTCCGATCT-3′)] with the following conditions: 30 s at 98°C, followed by 12× cycles of [10 s at 98°C, 20 s at 65°C and 15 s at 72°C], and finally 2 min at 72°C. The 200-bp PCR product was gel-extracted from a 2% TBE/agarose gel and isolated using a PureLink Quick Gel Extraction Kit (Invitrogen; K210012). 400 ng of pTSS-MPRA-Empty or pLenti-TSS-MPRA-Empty plasmid was digested with BsaI or BsmBI-v2, respectively, in 20 μl [1 μl BsaI (10 U), 2 μl CutSmart Buffer (NEB) or 1 μl BsmBI-v2 (10 U), 2 μl NEB3.1 (NEB)], respectively, at 37°C for 1 h. Linearized plasmid was gel-extracted and cleaned up using PureLink Quick Gel Extraction Kit (Invitrogen; K210012). Amplified oligo library was Gibson-assembled into cut plasmid using NEBuilder HiFi DNA Assembly Master Mix (NEB) with a 5-fold molar excess of the library at 50°C for 1 h in 4 μl total volume.

***Supplementary Note 1:*** *The amount of insert oligonucleotide pool used as starting material for PCR depends on the sequence complexity and size of the pool. The 2000-insert pool analyzed here is complex enough and small enough that the cloning procedure described is sufficient to generate a plasmid library pool that faithfully represents the diversity of the synthesized oligonucleotide pool. However, when amplifying large, low-complexity oligonucleotide pools (containing e.g. large numbers of sequences that differ only in a handful of positions, such as when mutating transcription factor motifs) (e.g. 18 000 oligonucleotides), with increasing PCR cycles and DNA amounts, partially elongated PCR products accumulate that can cross-hybridize to highly similar oligonucleotides. Elongation of these cross-hybrids leads to chimera formation, where the upstream sequence does not match the downstream barcode of the original oligonucleotides. This occurs more frequently at high DNA concentrations, likely when the PCR reaction goes into saturation early. Therefore, when amplifying larger oligo pools with many similar sequences, it is recommended to either start with much less template, e.g. 10 pg oligo pool/50 μl reaction and a single-step or two-step PCR, or emulsion PCR* (36). *Either approach lowers the starting concentration of the PCR template and will require scaling up the PCR volume to generate sufficient amounts of DNA for cloning plasmid libraries with good representation of the synthetic oligo pool. In either case, we recommend confirming a low chimera level in the cloned oligo pool plasmid library by paired-end sequencing and aligning the cloned sequences to the original sequences before proceeding*.

#### 350-bp insert library cloning

One-hundred nanograms of a 350-nt DNA oligonucleotide pool containing 500 insert/barcode combinations (IDT) (Supplementary Table S2) were PCR-amplified in 50 μl volume [25 μl NEBNext Ultra II Q5 Master Mix (NEB), 0.25 μl 100 μM pMPRA1-LH (5′-GGTAACCGGTCCAGCTCA-3′), 0.25 μl 100 μM pMPRA1-RH (5′-CGTGTGCTCTTCCGATCT-3′)] with the following conditions: 30 s at 98°C, followed by 15x cycles of [10 s at 98°C, 20 s at 65°C and 15 s at 72°C], and finally 2 min at 72°C. The PCR product (350 bp) was run on a 2% TBE/agarose gel, then gel-extracted and cleaned up using PureLink Quick Gel Extraction Kit (Invitrogen; K210012). Four-hundred nanograms pTSS-MPRA-Empty plasmid were digested with BsaI in 20 μl [1 μl BsaI, 2 μl CutSmart Buffer (NEB)] at 37°C for 1 h. Cut plasmid was gel-extracted and cleaned up using PureLink Quick Gel Extraction Kit (Invitrogen; K210012). Amplified library was Gibson-assembled into cut plasmid using NEBuilder HiFi DNA Assembly Master Mix (NEB) with a 5-fold molar excess of the library at 50°C for 1 hour in 4 μl total volume (higher complexity library assembly should be carried out in higher volumes).

#### 450-bp, 700-bp and 950-bp insert cloning

Each sequence was PCR-amplified from K562 genomic DNA in 50 μl volume [25 μl NEBNext Ultra II Q5 Master Mix (NEB), 0.25 μl 100 μM Fwd Primer (see Supplementary Table S3), 0.25 μl 100 μM Rev Primer (see Supplementary Table S3), 40 ng K562 genomic DNA] with the following conditions: 1 minute at 98°C, followed by 12× cycles of [10 s at 98°C, 30 s at 70°C and 30 s at 72°C], and finally 2 min at 72°C. PCR product was isolated with 10% (w/v) PEG 8000/SpeedBeads [2 μl SpeedBeads E3 (Cytiva, cat# 65152105050250) + 48 μl 20% (w/v) PEG 8000], beads were washed 2× with 80% ethanol and air-dried at RT for 12 min, and DNA eluted by resuspending in LoTE [1:4-diluted Tris-EDTA buffer pH 7.5]. Four-hundred nanograms pTSS-MPRA-Empty plasmid were digested with BsaI in 20 μl [1 μl BsaI, 2 μl CutSmart Buffer (NEB)] at 37°C for 1 h. Cut plasmid was gel-extracted and cleaned up using PureLink Quick Gel Extraction Kit (Invitrogen; K210012). Amplified sequences were Gibson-assembled into cut plasmid using NEBuilder HiFi DNA Assembly Master Mix (NEB) with a 2.5-fold molar excess of the library at 50°C for 1 h in 4 μl total volume.

#### Transformation

NEB Stable (lentivirus backbone) or NEB 5-alpha (plasmid backbone) chemically competent E.coli cells (50 μl; NEB C3040 or C2987, respectively) (see also Supplementary Note 2) were transformed with 1 μl Gibson assembly reaction according to manufacturer instructions and grown for 1 h in 950 μl outgrowth medium (SOC or Stable medium). Plasmid pool-containing cells (300 μl of SOC/Stable culture) were expanded in 200 ml LB Broth (100 μg/ml carbenicillin) for 16–18 h at 37°C (5-alpha) or 30°C (Stable) at 225 RPM and plasmids isolated with a PureLink HiPure Plasmid Maxiprep Kit (Invitrogen; K210006).

***Supplementary Note 2:*** *For higher complexity libraries we recommend using electroporation to introduce your plasmid constructs into bacteria. Briefly, we thaw ElectroMAX DH5a-E or ElectroMAX Stable electrocompetent cells (Invitrogen) on ice for 5 min, mix 2 μl of Gibson assembly product with 20 μl of competent cells in a cold 1.5 ml microcentrifuge tube, then transfer to a cold 0.1 cm electroporation cuvette. Electroporation is done using a BioRad GenePulser II at 1.2 kV (Stable) or 1.8 kV (DH5a-E), 200 Ω, and 25 μF. After electroporation, 1 ml of SOC medium is added to the cuvette immediately, mixed by slowly pipetting up and down, then transferred to a 14-ml snap-cap tube and incubated at 30°C (Stable) or 37°C (DH5a-E) for 1 hour. Plasmid pool-transformed cells (300 μl of SOC/Stable culture) are then expanded in 200 ml LB Broth (100 μg/ml carbenicillin) for 16–18 h at 37°C (5-alpha) or 30°C (Stable) at 225 RPM and plasmids isolated with a PureLink HiPure Plasmid Maxiprep Kit (Invitrogen; K210006)*.

#### Library DNA insert sequencing

One nanogram of plasmid library was first PCR-amplified in 50 μl volume [25 μl NEBNext Ultra II Q5 Master Mix (NEB), 0.25 μl 100 μM P5-1 Primer (5′-CTACACGACGCTCTTCCGATCT GGTAACCGGTCCAGCTCA-3′), 0.25 μl 100 μM P7 Primer (unique indexed TruSeq D701-D710 primer for each sample)] with the following conditions: 2 min at 98°C, followed by 8x cycles of [10 s at 98°C, 20 s at 65°C and 20 s at 72°C], and finally 2 min at 72°C. PCR product was isolated with 10% (w/v) PEG 8000/SpeedBeads [2 μl SpeedBeads E3 (Cytiva, cat# 65152105050250) + 48 μl 20% (w/v) PEG 8000], beads washed 2× with 80% ethanol and air-dried at RT for 12 min, and DNA eluted by resuspending in LoTE [1:4-diluted Tris-EDTA buffer pH 7.5].

PCR product was quantified by Qubit HS DNA kit (Thermo) and diluted to a concentration of 1 ng/μl. To add primer landing sites for the P5 flow cell primer, 1 ng PCR product was amplified in a second 50-μl PCR reaction with TruSeq P5/P7 primers [25 μl NEBNext Ultra II Q5 Master Mix (NEB), 0.25 μl 100 μM P5-2 Primer (TruSeq D50* indexes), 0.25 μl 100 μM P7 Primer (TruSeq D701-D710 indexes)] with the following conditions: 30 s at 98°C, followed by 12× cycles of [10 s at 98°C, 20 s at 68°C and 20 s at 72°C], and finally 2 min at 72°C. Libraries were run on 2% TBE/agarose gel, gel-extracted and cleaned up using PureLink Quick Gel Extraction Kit (Invitrogen; K210012). Libraries were paired-end sequenced for 64 cycles (Read 1) and 20 cycles (Read 2) on an Illumina NextSeq 500.

#### Plasmid electroporation

Three million K562 cells per electroporation were centrifuged (6 min, 300 g, RT), supernatant discarded and the cell pellet resuspended and centrifuged twice in 10 ml reduced-serum Opti-MEM medium (Thermo) with the same centrifugation conditions. Cells were then resuspended in 200 μl Opti-MEM per electroporation and mixed with 10 μg of plasmid insert library. The Opti-MEM + insert library was deposited into a 4 mm cuvette and electroporated with a BioRad GenePulser Xcell with a single exponential decay pulse at 200 V, 1000 uF, ∞ Ohm resistance. Electroporated cells were quickly transferred into 2.8 ml of 10% FBS/RPMI 1640 and incubated at 37°C, 100% humidity, 5% CO_2_ for 24 h.

#### Lentiviral transduction

HEK293T cells were cultured in 6-well plates that had been coated with poly-d-lysine for 20 min at room temperature (RT), washed 4× with 1 ml PBS and filled with 1 ml of media to avoid drying out the plates. The day before transfection, 600k HEK293T cells were plated per well. HEK293T cells were transfected the next morning with Lipofectamine 3000 using insert library, pMD2.G, and psPAX2 plasmids at a ratio of 4:1:3 (1.5 μg of insert library). Media was changed ∼18 h after transfection. Viral supernatant was collected in 15 ml centrifuge tubes 48 h after replacing media and replaced with 3 ml of fresh media per well and stored at 4°C. Viral supernatant was collected a second time 24 h later (72 h after first media change). Twenty-four h before transduction, 200k K562 cells per well were plated in a 6-well plate in 3 ml of 10% FBS/IMDM.

Lentiviral supernatant was centrifuged at 1000 g for 5 min and the supernatant was passed through a 0.45 μm syringe filter to remove cell debris. K562 cells were transduced by adding 1.5 ml of unconcentrated viral supernatant and 8 μg of polybrene to each well, and spinoculating at 600 × g for 90 min at 37°C. After spin-fection, 1.5 ml of fresh media was added to each well and the cells cultured for 24 h in a humidified incubator at 37°C, 5% CO_2_. The virus-containing media was replaced with 3 ml of fresh media and cells were cultured for another 48 h before being collected for downstream experiments.

#### RNA extraction

Cells were collected and resuspended in 250 μl ice-cold PBS. Trizol LS (750 μl) was added to each sample and incubated for 5 min at RT. 230 μl of chloroform-isoamyl alcohol was added to each and incubated for 5 min at RT. Samples were then centrifuged for 15 min (13 000 × g, 4°C). Supernatant containing the RNA was transferred to a new 1.5 ml tube and 1 μl of GlycoBlue (Thermo) + 1/10th volume of 3 M sodium acetate was added. After mixing quickly, 1× volume of isopropanol was added and inverted 10×, then spun down briefly. Samples were incubated overnight at −20°C, then centrifuged for 30 min (25 000 × g, 4°C) the next day. Supernatant was discarded, RNA pellet was washed 1× with 75% ethanol, and then air-dried for 3 min at RT. The RNA pellet was resuspended in 30 μl of TLoET [0.05% Tween, 0.1 mM EDTA, 10 mM Tris pH 7.5].

#### DNA extraction

The lower Trizol phase from RNA extraction was spun down at 12 000 × g for 10 min and any remaining aqueous layer overlaying the interphase was removed. DNA was precipitated by adding 400 μl of 100% ethanol to each sample, inverting tubes 10 times, incubating samples at RT for 3 min and 5-min centrifugation at 2000 × g, 4°C. The interphase and phenol layer were removed and the DNA pellet was washed by incubation with 1 ml of 0.1 M sodium citrate in 10% ethanol (pH 8.5) for 30 min. The pellet was centrifuged for 5 min at 2000 × g, 4°C and the supernatant was discarded. The sodium citrate wash was repeated once before washing the pellet in 1.5 ml 75% ethanol for 20 min. The pellet was centrifuged for 5 min at 2000 × g, 4°C, the supernatant was discarded, and the DNA pellet was air-dried for 10 min. The pellet was dissolved in 100 μl 8 mM NaOH and the solution centrifuged for 10 min at 12 000 × g, 4°C to remove insolubles. The supernatant containing the DNA was transferred to a clean tube and its pH was adjusted to a pH of 8.3–8.4 using HEPES pH 8.0.

#### Capped MPRA 5′ RNA-seq

Total RNA (5–15 μg) in 15 μl TLoET was denatured at 75°C for 90 s, and rapidly chilled on wet ice. To dephosphorylate non-5′-capped 5′ ends and simultaneously degrade carried-over DNA, we added 35 μl calf intestinal phosphatase mix [25.25 μl 0.05% Tween 20, 5 μl CutSmart Buffer (NEB), 0.75 μl SUPERase In™ RNase Inhibitor (20 U/μl, Thermo Fisher Scientific), 0.5 μl RQ1 DNase (Promega), 2 μl Quick CIP (NEB)], mixed well and incubated at 37°C for 1 h. To improve 5′ enrichment, RNA was briefly denatured a second time at 75°C for 30 s, quickly chilled on ice for 2 min and incubated at 37°C for an additional 30 min. Following dephosphorylation of uncapped RNAs, reactions were inactivated by mixing with 500 μl Trizol LS, then 140 μl TLoET and 140 μl CHCl_3_ + IAA (24:1, Sigma) were added. Samples were vortexed vigorously and centrifuged for 10 min at 12 000 g, RT (21°C). Following phase separation, the upper layer was transferred to a new tube and RNA precipitated by mixing with 1/10 volume 3 M NaOAc pH 5.5 and 1 volume 100% isopropanol. The mixture was incubated at −20°C for at least 20 min to overnight, and the RNA precipitated by centrifuging for 30 min at 21 000 g, 4°C. The supernatant was discarded, remaining liquid collected by brief centrifugation and removed prior to washing the RNA pellet with 400 μl 75% EtOH by inversion. Samples were centrifuged for 5 min at 21000 g, 4°C, the supernatant completely removed and the pellet air-dried at RT for 5 min.

RNA pellets were resuspended in 5 μl TLoET, denatured at 75°C for 90 s, then quickly chilled on ice. RNA was decapped by adding 10 μl Decapping Master Mix [3.25 μl 0.05% Tween, 1.5 μl T4 RNA Ligase Buffer (NEB), 4 μl PEG 8000 (50%), 0.25 μl SUPERase In RNase Inhibitor (Thermo) and 1 μl RppH (NEB)] and incubating at 37°C for ≥1 h. An RNA adapter was ligated to the decapped, monophosphorylated 5′ ends by adding 10 μl L1 mix [1 μl T4 RNA ligase Buffer, 2 μl 10 mM ATP, 1 μl 10 μM NEBNext 5′ SR Adaptor for Illumina (NEBNext Multiplex Small RNA Library Prep Set for Illumina (NEB)), 5 μl PEG and 1 μl T4 RNA Ligase 1 (NEB)] and incubation at RT (21°C) for 2 h. The ligation product was isolated by Trizol cleanup as described above.

RNA pellets were resuspended in 7 μl of Annealing Master Mix [1 μl of 20 µM RS2 primer (5′-AGCGGATAACAATTTCACACAGGA-3′), 2 μl 700 mM KCl and 4 μl TET (10 mM Tris pH 7.5, 1 mM EDTA, 0.05% Tween 20)] and incubated at 75°C for 90 s followed by 30 min at 56°C and then ice. Next, 13 μl RT Master Mix [7.5 μl 0.05% Tween, 1 μl reverse transcription buffer (500 mM Tris–HCl pH 8.3, 30 mM MgCl_2_), 2 μl 0.1 M DTT, 2 μl 10 mM dNTPs, 0.5 μl SUPERase In RNase Inhibitor and 1 μl Protoscript II (NEB)] were added and samples incubated at 50°C for 1 h.

MPRA transcript 5′ ends were PCR-amplified for 14 cycles in 50 μl volume [25 μl LongAmp Taq 2X Master Mix (NEB), 2.8 μl 5 M Betaine (Sigma), 2 μl 10 μM NEBNext Index 1 Primer for Illumina (TruSeq R2-compatible), 0.2 μl 100 μM NEBNext SR Primer for Illumina] with the following conditions: 30 s at 948°C, followed by 15x cycles of [1 s at 94°C, 30 s at 63°C and 18-60 s at 70°C (depending on insert length)], and finally 5 min at 70°C. RNA was degraded by adding 1 μl 100 μg/μl DNase-free RNase A (Qiagen) and incubating for 15 min at 37°C. The PCR product was purified by incubating for 10 min at RT with SpeedBeads and 12.2% PEG 8000/1.38 M NaCl final [added 2 μl Sera-Mag SpeedBeads (Cytiva), 34 μl 5 M NaCl, 37.5 μl 40% PEG], beads were collected on a magnet, washed 2x with 80% ethanol and air-dried at RT for 12 min. DNA was eluted by resuspending the beads in 10 μl LoTE [1:4-diluted Tris-EDTA buffer pH 7.5]. DNA size distribution was checked by running the DNA on a 10% PAGE/TBE gel (Novex) and staining with SYBR Gold (Invitrogen). DNA larger than 125 bp up to a size of (insert length + 121 bp (adapter size)) were size-selected and isolated using Zymo Clean & Concentrator-5 columns (Zymo Research) and sequenced paired-end on an Illumina NextSeq 500.

An updated step-by-step TSS-MPRA protocol is available at protocols.io (dx.doi.org/10.17504/protocols.io.kqdg3p7kzl25/v1).

### Data analysis

#### Mapping of TSS-MPRA-seq data

A custom genome index made up of all of the sequences in the insert library was generated using STAR version 2.7.9a as follows: ‘STAR –runMode genomeGenerate –genomeDir < output_directory> –genomeFastaFiles < insert_library_fasta.fa> –genomeSAindexNbases 6'.

Paired-end reads were mapped to the custom genome index using STAR as follows: ‘STAR –genomeDir < reference_genome_directory> –readFilesIn < read1_fastq.fq> <read2_fastq.fq> –outFileNamePrefix < output_prefix> –alignEndsType Extend5pOfRead1 –outSAMmultNmax -1 –outReadsUnmapped Fastx –alignEndsProtrude 50 ConcordantPair –seedMultimapNmax 1000000'.

#### Mapping of csRNA-seq data

csRNA-seq reads were mapped to the hg38 genome using STAR as follows: ‘STAR –genomeDir < hg38_genome_directory> –readFilesIn < fastq.fq> –outFileNamePrefix < output_prefix> –outSAMstrandField intronMotif –outMultimapperOrder Random –outSAMmultNmax 1 –outFiltermultimapNmax 10000 –limitOutSAMoneReadBytes 10000000'.

#### Quantification

TSS frequencies for both TSS-MPRA and csRNA-seq data were calculated by counting the number of times the 5′ end of read 1 overlapped each nucleotide. Total RNA abundance was calculated as the total number of TSS-MPRA-seq reads (read 2) that mapped to the unique barcode associated with each sequence. DNA abundance was calculated as the total number of DNA-seq reads (read 2) that mapped to the unique barcode associated with each sequence.

#### RNA abundance normalization

After quantification, total RNA and DNA abundances were transformed into counts per million reads sequenced (CPM) to account for differences in sequencing depth. CPM-transformed insert RNA values were then normalized by dividing RNA abundance by the corresponding DNA abundance (RNA/DNA), i.e. transcript levels of each insert were expressed as the ratio between its total CPM-transformed RNA abundance divided by total CPM-transformed DNA abundance.

#### Track visualization

TSS frequencies for all inserts were quantified as described above, and single-nucleotide bedgraph files were generated using a custom script (see GitHub). All bedgraph files were visualized using CoolBox (37).

#### Barcode replicate variance analysis

To measure the degree of dispersion in transcription levels of barcode replicates we zero-centered transcription levels by subtracting the median Log2 DNA-normalized RNA count of each insert group (each insert group includes 4 inserts made up of barcode replicates) from each insert's RNA count value within the group. The standard deviation of the resulting value distribution of all zero-centered insert values was then calculated and outliers determined as those inserts that had DNA-normalized transcription levels at least 3 standard deviations from the mean of the insert group.

#### Calculating WIP similarity scores

The windowed initiation profile (WIP) scoring metric quantifies the dissimilarity between two transcription initiation patterns by comparing the position-specific initiation signal between two data sets. The algorithm employs a sliding window approach to scan across two given TSS distributions and then sums up the absolute differences in relative initiation signal for each window. This is done for increasing window sizes, starting with a size of 1 (here: ranging from 1 to 5 base positions). The sum of the combined differences at each window size multiplied by the window size makes up the WIP score, which sensitively quantifies the degree of divergence between two TSS profiles (Supplementary Figure S4).

Computationally, TSS distributions are represented as 1D arrays of identical length. Each value in the array represents the position-specific raw signal (here: 5′ RNA-seq sequencing reads that start at that nucleotide position within the TSS distribution). Raw values are transformed to counts per million (CPM) to account for sequencing depth differences between experiments. To ensure that comparisons between arrays are independent of overall signal intensity, arrays are further normalized so that all values within the array sum up to one. Dissimilarity between two 1D arrays is computed by sliding a window of length *k* across both arrays, calculating the absolute differences in normalized array values at each window and adding them to a running *k-diff* score. Each final *k-diff* score is multiplied by the length of the window (*k*) and then added to a *diffsum* score. This process is repeated a total of *k* times (*k* = 5 in this manuscript). The final *diffsum* score computed after this iterative process is equivalent to the WIP score.

WIP scores are a useful metric to quantify how different two (experimentally defined) TSS profiles are, but by themselves are not sufficient to determine whether two distributions are significantly different in relation to the other experimental results. Therefore, to determine whether two TSS distributions are significantly different from each other, we used csRNA-seq replicate datasets as ground truth to fit a novelty detection model. Our assumption was that there should be some minimal variation in TSS distribution between identical gene promoters in any two replicates, and that any large differences outside of that variation could be considered as outliers. We first examined the 300 bp surrounding annotated transcription start sites (−150/+150 from the TSS) and quantified TSS frequencies as described above. We calculated WIP scores for identical gene promoters between two replicates (after filtering out promoters with < 25 reads). We then trained a one-class support vector machine (OCSVM) model (38) on the WIP scores and mean quantile-normalized RNA abundance counts from each promoter replicate. Using this novelty detection model we can determine whether a new data point (WIP score and mean quantile-normalized RNA abundances between any two TSS distributions) is a member of a specific class or not (i.e. significantly different from the normal variation observed in replicate csRNA-seq datasets or not). The output of our model is the classification score of a new data point being an outlier (where 0 is completely within the normal variation observed between replicate datasets, and 1 is completely outside), wherein data points closer to 1 represent two significantly different TSS profiles. For most purposes in this manuscript, unless stated otherwise, we assume that any two TSS profiles with an outlier score greater than 0.5 are considered to be significantly different.

#### Initiation pattern reproducibility analysis

To calculate the reproducibility of TSS-MPRA initiation patterns across replicates, we compared the WIP scores of three groups: (A) scores between identical promoters in two TSS-MPRA replicate experiments (true positives), (B) average scores between TSS-MPRA barcode replicates (quadruplicates within each experiment) and (C) average scores between non-barcode-replicates (all against all within experiment) (true negatives).

#### WIP score vs earth mover's distance comparisons

We first calculated the WIP scores and Earth Mover's Distance between each insert in the TSS-MPRA against all 2000 inserts (500 sequences barcoded 4-fold) within the same experiment, in two replicate experiments, for a total of 4000 separate comparisons. We then ranked the dissimilarity score lists for each sequence against all sequences in each experiment and counted the number of times that a given sequence's barcode replicates appeared in the top 4 most similar (lowest) scores in each list. To calculate Earth Mover's Distance, we used three different methods: (i) EMD using the Python Optimal Transport (POT) library (https://pythonot.github.io/), (ii) EMD using the pyEMD library (https://github.com/laszukdawid/PyEMD), EMD using Scipy's Wasserstein Distance function and (iii) a custom function. We observed that EMD calculations between POT, pyEMD and Scipy vary widely, while results from Scipy and our custom EMD calculation were identical.

#### OCSVM model accuracy analysis

To measure the accuracy of our OCSVM model, we worked under the assumption that there should be little differences between the WIP scores of identical promoters in replicate csRNA-seq and TSS-MPRA experiments. WIP scores between csRNA-seq vs TSS-MPRA were calculated for the same promoters in each replicate separately and used as input, along with the median RNA expression of each promoter, for our OCSVM model. We then defined the accuracy of our model as the frequency in which the same promoter is labeled differently (e.g. inlier in replicate 1, but outlier in replicate 2) by the model.

#### Sequencer size bias normalization

To account for the bias in sequencing shorter inserts versus longer inserts we fit a spline to the discrete NextSeq size bias profiles from Gohl *et al.* (39) and used the interpolated size bias as scaling function to normalize the read counts at each bp for track visualization.

#### Calculating focus ratios

To quantify the spread of a TSS profile, focus ratios are calculated as the number of reads within 10 bp of a TSS location of interest divided by the total number of reads within a region (here: TSS-MPRA insert size). Focus ratios range from 0 to 1 where 0 indicates an extremely dispersed TSS profile while 1 indicates a highly focused (single-nucleotide) TSS profile.

## RESULTS

### TSS-MPRA, a simplified TSS-aware MPRA protocol with low input requirements

Different transcription regulatory mechanisms are associated with distinct promoter transcription initiation patterns (7,16,17). Yet, despite widespread use of MPRAs to study transcription regulation for over a decade, it remains unclear how well transcription initiation patterns of promoters in MPRA reporter constructs recapitulate those of the endogenous promoters *in vivo*. Only a single study has attempted to compare transcription initiation *in vivo* with initiation at corresponding inserts in an MPRA. Arnold *et al.* (6) analyzed averaged initiation signals of thousands of *drosophila* genome fragments in STAP-seq and found that the main averaged initiation site aligned with gene TSS annotated in FlyBase. While this indicates that the averaged main TSS of the majority of promoters in MPRAs coincides with the main TSS positions of genes *in vivo*, this analysis does not address whether the initiation patterns of individual MPRA inserts recapitulate the corresponding initiation patterns—and hence regulatory mechanisms—of the corresponding endogenous sites.

**Figure 1.**
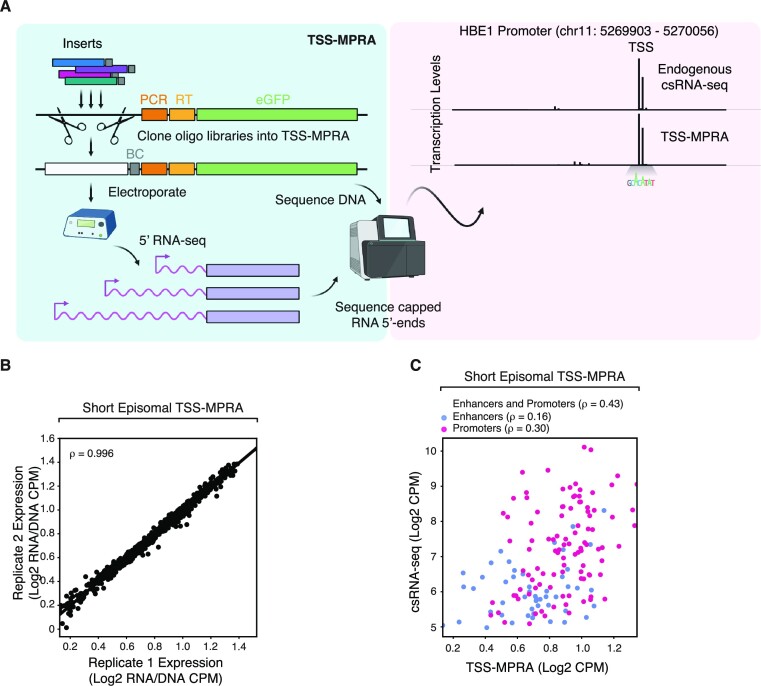
TSS-MPRA of synthetic regulatory sequences mirrors the vast majority of endogenous initiation patterns and transcription levels. (**A**) Schematic of TSS-MPRA. Transcription activity and location of transcription initiation is determined by 5′ RNA-seq of reporter transcripts initiating within synthetic DNA inserts cloned into reporter plasmids and electroporated into cells. Histograms on the right show cumulative DNA-normalized number of sequence tags aligning to each base position of a 153-bp region surrounding the human HBE1 promoter in K562 cells. Capped reporter transcripts are drawn in purple. RT: reverse transcription primer landing site. BC: barcode sequence. (**B**) Spearman's correlation of DNA-normalized RNA levels of all inserts of two replicate episomal TSS-MPRA experiments. (**C**) Correlation between the transcriptional signal of 250 genomic DNA inserts in *epi-short* TSS-MPRA and csRNA-seq of the corresponding endogenous loci. TSS-MPRA inserts were chosen randomly from locations exhibiting transcription activity as measured by csRNA-seq. Spearman's correlation of TSS-MPRA and csRNA-seq transcription levels of all regulatory sequences, or of promoters (red) or enhancers (blue, outside of a ± 2-kb window of RefSeq-annotated promoters). Regions were chosen to cover a wide range of transcription levels and initiation patterns. Each dot represents the relative transcript levels observed in each assay as total normalized read counts of all transcripts aligning to each region. TSS-MPRA RNA read counts were normalized by the corresponding plasmid DNA read counts.

To determine TSS profiles of synthetic DNA inserts and assess promoter transcription initiation fidelity of individual inserts relative to the genome in an MPRA, we devised an MPRA insert barcoding scheme with 11-mer barcodes immediately downstream of the genomic sequence that are synthesized together as part of one oligonucleotide (Supplementary Figure S1A). Cloned into a reporter plasmid, these barcodes are transcribed as part of the 5′ UTR of the reporter transcript. This enables interrogating the effect of DNA sequence variants upstream of the reporter transcript on the location of transcription initiation (Figure 1, Supplementary Figure S1C). A barcoded variant of STAP-seq with an almost identical barcoding approach was published while this manuscript was under review (8) (Supplementary Figure S1B). This type of 5′ reporter transcript barcoding is broadly similar to two previously published methods (25,26) but with important differences. Lubliner et al. encoded barcodes as silent mutations in the N-terminal 12 codons of the reporter gene, taking up a 36-nt portion of each designed oligonucleotide insert used to clone each promoter construct. Together with a 10-nt A-rich tract, this limited the effective insert size of the promoter portion of each synthetic 200-nt oligonucleotide to 118 nucleotides (Supplementary Figure S1B). In contrast, the MASTER method (26,32) uses randomized oligonucleotides (7–10 nt random sequence at the TSS of a minimal promoter and 15–20 nt random sequence downstream of the TSS) (Supplementary Figure S1B). This provides full coverage of all possible sequence variants but is only feasible for short stretches of random sequence (at 100x RNA-seq coverage, the diversity of e.g. a 15-mer random region (1.07 × 10^9^ × 100 = 107 × 10^9^) would already exceed the capabilities of the most recent Illumina sequencer (52 × 10^9^ reads)) and does not permit introducing defined nucleotide changes throughout larger inserts.

For high-throughput cloning of DNA inserts, we generated promoter-less derivatives of the commercial pGL4.10 reporter plasmid (pTSS-MPRA for episomal TSS-MPRA) and the pLS-SceI lentiviral transfer plasmid (Gordon et al. 2020) (pLenti-TSS-MPRA for chromatinized TSS-MPRA) for Gibson assembly. Both plasmids contain eGFP reporter genes with upstream primer landing sites for reverse transcription and Illumina sequencing, and two type IIS restriction sites flanked by homology cloning arms for linearization and insert cloning by Gibson assembly (Supplementary Figure S1A and Materials and Methods). TSS-MPRA uses a designed 18-nt sequence as left and 18 nt of the 3′ end of the Illumina TruSeq Read 2 primer sequence as right homology arm to maximize the usable insert size. Compared to STAP-seq, which directly incorporates Illumina TruSeq Read 1/Read 2 primer sequences for Gibson assembly and DNA sequencing library construction (Supplementary Figure S1B) but requires 25-nt homology arms due to the inverted terminal repeat generated by the identical 13-nt 3′ ends of these sequences, this increases the usable insert size by 14 nt.

To enable performing TSS-MPRA from limited amounts of RNA after e.g. lentiviral transduction, we devised a library prep procedure similar to the one used by Lubliner *et al.* (40), which generates a sequencing library from total RNA by ligating a 5′ adapter selectively to capped RNAs followed by reporter-specific reverse transcription and amplification (Supplementary Figure S1C). This allows TSS-MPRA to be performed from 5–10 μg total RNA, or 2–3 million cells, which compares favorably to the ≥ 40 million cells required to isolate the 10 μg of mRNA starting material needed for STAP-seq library preparation (7).

### Moderate agreement between MPRA-derived and endogenous initiation profiles and transcription levels of human promoters in an episomal MPRA setting

To test TSS-MPRA performance, we designed a library of 500 human promoter and enhancer sequences surrounding active TSSs as determined by csRNA-seq (5) in K562 erythroleukemia cells. Half of the sequences were randomly chosen regulatory elements covering a range of transcriptional activities and TSS profile shapes, the other half were sequences designed to test the effect of mutating transcription factor or core promoter motifs and single nucleotide polymorphisms (SNPs) on initiation patterns and transcription levels. Each 200-bp synthetic sequence in the library contained a 153-bp genomic sequence insert (from −110 to + 43 bp around the most active TSS within a given window), a unique 11-mer barcode (41) as well as flanking 18-bp homology arms for directional cloning via Gibson assembly (Figure 1A). To account for potential barcode-specific effects, each sequence was barcoded 4-fold redundantly, for a total of 2000 inserts. We refer to this oligonucleotide pool as *epi-short* throughout the text.

*Epi-short* inserts were PCR-amplified and shotgun-cloned into the pTSS-MPRA plasmid. The plasmid pool was electroporated into K562 cells in duplicate and electroporation efficiency/viability verified by fluorescence microscopy (>80% cells eGFP + with ∼60% cell viability as measured by trypan blue exclusion). Total RNA was isolated after 24 h, and reporter-specific 5′ RNA-seq libraries were prepared from 10 μg total RNA. Briefly, non-capped RNA was dephosphorylated with calf intestinal phosphatase, mRNAs were decapped with RppH 5′ pyrophosphohydrolase to leave a 5′ monophosphate, and an Illumina-compatible 5′ RNA adapter was ligated to these previously capped RNA 5′ ends. Plasmid-derived transcripts were selectively reverse-transcribed with a reporter transcript-specific reverse transcription primer and the cDNA PCR-amplified with Illumina-compatible primers (Supplementary Figure S1C). In parallel, we generated input DNA-seq libraries from DNA isolated from the same cell lysates by PCR to normalize TSS-MPRA RNA signal to the plasmid levels in the cells. After sequencing, 96% of TSS-MPRA RNA and 99% of DNA reads aligned to library inserts, with 95% of inserts exhibiting quantifiable levels of transcription (defined as having > 25 mapped reads). As exemplified for the sequence surrounding the HBE1 promoter locus, TSS-MPRA can reliably capture transcription initiation frequencies and locations from DNA inserts (Figure 1A). RNA, DNA and DNA-normalized RNA levels between replicate experiments were highly reproducible (ρ > 0.99 for both; Figure 1B, Supplementary Figure S2), with little transcript-level variance between barcode replicates within insert groups (SD = 1.4-fold around the median; see Methods) (Supplementary Figure S3).

Next, we determined how promoter activity measured by TSS-MPRA compares to a previously published non-TSS-aware promoter MPRA that employs 3′ barcoding, SuRE-seq (42). While promoters in a reporter plasmid lack their native chromatin environment, the only previous comparison of SuRE-seq and nascent transcription initiation data measured by GRO-cap has shown that MPRA data replicates endogenous promoter activity surprisingly well (*r* = 0.43) (42)). The transcription levels of the 250 random regulatory elements in our *epi-short* plasmid pool measured by TSS-MPRA and genomic transcription initiation as measured by csRNA-seq correlated to the same degree (Spearman ρ = 0.43, Peason *r* = 0.44; Figure 1C), suggesting that 5′ barcoding of reporter transcripts yields comparable information about promoter activity as the more widely used 3′ barcoding MPRA strategy.

The randomly chosen inserts of the *epi-short* pool contain genomic loci annotated as either promoters or putative enhancers. Segregating TSS-MPRA results by promoter and enhancer annotation revealed that promoters had higher levels of correlation with csRNA-seq than enhancers (Promoter ρ = 0.30, Enhancer ρ = 0.16; Figure 1C).

Taken together, our data indicate that TSS-MPRA recapitulates transcription levels of promoters to a similar degree as a published, non-5′-aware MPRAs, while proper enhancer transcription may be more frequently dependent on additional sequence features not encoded within the short, 153-bp pieces of genomic sequence tested.

### Windowed initiation profiling scores sensitively quantify differences between TSS profiles

Next, we set out to compare TSS-MPRA and endogenous transcription initiation profiles. Historically, two different approaches have been used to characterize transcription initiation patterns: promoters are either classified based on the ‘shape’ of their TSS distribution using rule-based methods, or pairwise comparisons are carried out using a dissimilarity metric to quantify the difference between the TSS distributions of individual promoters. The first global analysis of TSS patterns by Carninci et al. used a rule- and quantile-based classification scheme on CAGE data to stratify promoter TSS patterns into four classes: single-peak patterns were classified as ‘sharp’, while broad TSS patterns were further separated into three subclasses (without peak, with one dominant peak or multimodal) (11). While this type of analysis helps cluster promoters with similar distributions together, it does not offer an easy way to quantify how different the TSS patterns of individual promoters are from one another. In 2011, Zhao et al. extended Carninci's classification system by modifying a non-parametric distance metric based on the Minimum Difference of Pair Assignments (MDPA) (43) to measure how dissimilar one TSS distribution is from another, which they called the Generalized Minimum Distance of Distributions (GM-distance) (44). This approach offers a quantifiable metric that can be used to describe how similar or different the transcription initiation patterns of two promoters are. Since then, the majority of analyses comparing TSS distributions have used some version of MDPA or the similar Earth Mover's Distance (EMD) (45–48).

We initially benchmarked several distance similarity metrics, including EMD (45), GMDP (49), Pearson Correlation, Cosine Similarity, Dynamic Time Warp, and Root Mean Squared Deviation. However, none of them performed satisfactorily to capture the often subtle but clearly visible differences in TSS patterns. To more accurately assess the degree to which TSS-MPRA mirrors endogenous transcription initiation patterns, we developed a new metric called the ‘windowed initiation profile’ (WIP) score. WIP scores quantify the degree of dissimilarity between two initiation patterns by summing up the weighted differences in relative magnitude of the initiation signal at each nucleotide position within sliding windows of increasing size across a region. By summing up weighted differences at different scales, this approach takes the discontinuous, but hierarchical nature of transcription initiation patterns into account (Supplementary Figure S4). Benchmarking the WIP score against EMD, a popular metric used to compare TSS profiles (44,48), on TSS-MPRA replicate data, we found the WIP score metric to more frequently identify differently barcoded insert replicates within each MPRA experiment (Supplementary Figures S5–S7).

**Figure 2.**
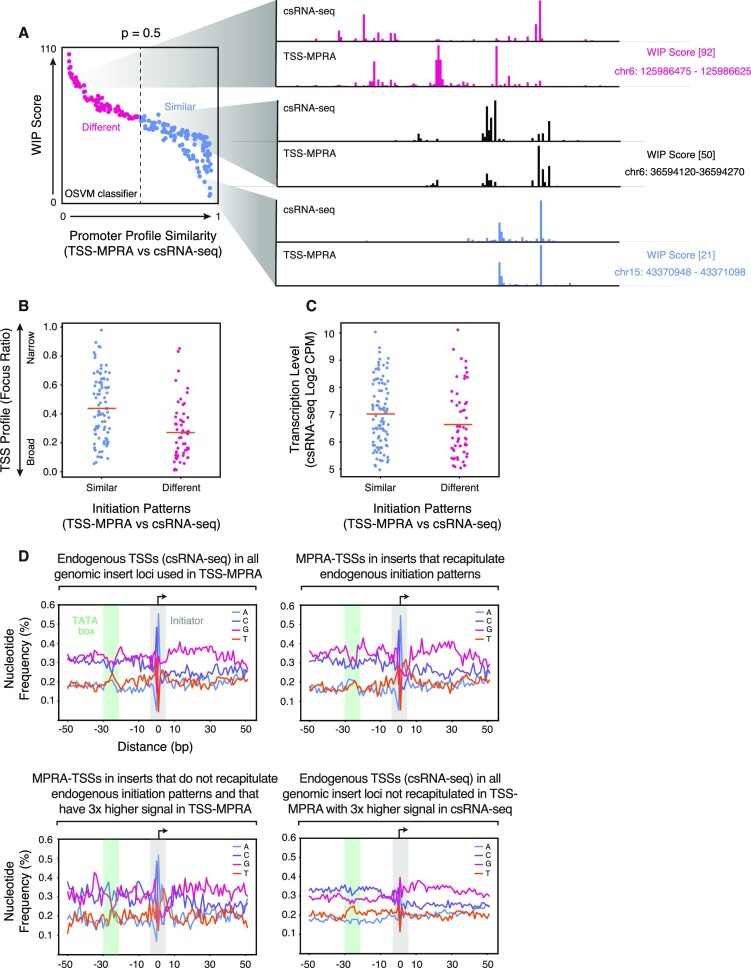
TSS-MPRA fidelity correlates with genomic TSS pattern width, transcription level, and presence of core promoter elements. (**A**) Schematic of the outlier detection model used to determine whether two TSS distributions are similar or not. Higher WIP scores (see methods for derivation) are indicative of more dissimilar initiation patterns. (**B**) TSS-MPRA preferentially recapitulates focused initiation patterns. Focus ratios (y-axis) of TSS-MPRA initiation patterns that are similar (blue) or dissimilar (red) to the corresponding endogenous initiation patterns as measured by csRNA-seq. Focus ratios of 0 indicate fully dispersed (broad) initiation patterns, while 1 indicates fully focused (sharp) patterns. (**C**) TSS-MPRA better recapitulates initiation patterns of more actively transcribed genomic regions. Endogenous locus transcription levels (csRNA-seq tag counts, y-axis) where TSS-MPRA initiation shape is similar (blue) or dissimilar (red) to the corresponding endogenous initiation pattern. (**D**) Strong transcription in the TSS-MPRA correlates with presence of TATA and Inr core promoter elements. Position-specific nucleotide frequencies (y-axis) (A: blue, C: purple, G: red, and T: orange) relative to each TSS in all TSS-MPRA inserts where: overall TSS-MPRA shapes of the inserts mirror the endogenous initiation patterns (I), or where insert TSS shapes do not mirror endogenous initiation patterns and either the respective TSSs within the overall TSS shape have 3x higher contribution to the overall signal of a given insert in TSS-MPRA data than in csRNA-seq (II), or the respective TSSs have 3× higher contribution to the overall signal of a given insert in csRNA-seq over TSS-MPRA data (III). The x-axis denotes the distance in bp from each TSS (bp 0).

While the WIP score provides an excellent metric for initiation profile dissimilarity, whether two TSS profiles can be considered ‘significantly’ different from each other depends on how replicable profile shapes are in general, which is dependent on signal and noise levels and overall assay variability. To determine when two TSS shapes should be considered ‘significantly’ different, we trained a novelty detection model based on a one-class support vector machine (OCSVM) on replicate genome-wide transcription initiation data set measured by csRNA-seq, using the WIP scores and average transcription levels for all RefSeq-annotated gene promoters to experimentally define dispersion between biological replicates (Figure 2A; see Materials and Methods). Based on the score distribution, we call TSS profile shapes different with an SVM outlier score of <0.5.

Applying WIP score analysis to TSS profiles of TSS-MPRA inserts and their corresponding endogenous profiles as measured by csRNA-seq, we found that 63% of the 250 randomly chosen regulatory elements in the *epi-short* pool recapitulated the corresponding endogenous transcription patterns. Analysis of the csRNA-seq TSS profiles of the 37% regulatory elements that did not recapitulate endogenous TSS profile shapes showed that they had more dispersed initiation patterns and lower average levels of transcription (Figure 2B, C).

To gain insights into the sequence features of the two different classes of inserts, we analyzed their nucleotide frequency distributions. Cumulative TSS-centered analysis of the nucleotide frequencies surrounding each TSS revealed that the TSSs within TSS-MPRA inserts that recapitulated endogenous TSS profile shapes exhibited nucleotide frequency patterns consistent with Initiator and upstream TATA box motif enrichment at 0 nt and −30 nt relative to the TSS, respectively (Figure 2D, upper right panel). These two core promoter elements are also enriched at regions surrounding endogenous sites of transcription initiation (Figure 2D, upper left panel) and are known to be associated with focused, high-level transcription initiation (12). Similarly, individual TSSs that were more active in TSS-MPRA inserts with TSS patterns different from the endogenous TSS profiles were also enriched for TATA and Inr-like nucleotide frequencies (Figure 2D, lower left panel). In contrast, individual TSSs that were more active in csRNA-seq than in TSS-MPRA and that were located in inserts that did not recapitulate the endogenous TSS profile in TSS-MPRA had an overall more pyrimidine-rich profile upstream, including at the TATA box location at −30 nt, and a bias for G as the first transcribed nucleotide, rather than the typical A nucleotide (Figure 2D, lower right panel).

We also found that the transcription levels of inserts with lower WIP scores (i.e. with initiation patterns more similar to the endogenous locus) correlated better with endogenous transcription levels than sequences with higher WIP scores (Supplementary Figure S8), indicating a relationship between TSS profile shape and transcription levels. While the majority of sequences in our TSS-MPRA drive accurate initiation patterns, a significant fraction of tested sequences gave rise to divergent TSS shapes. These sequences likely use alternative regulatory mechanisms when in the context of a reporter plasmid, and mechanistic studies of their function in a reporter may lead to misleading results.

### Increasing the size of oligonucleotide inserts in the MPRA does not lead to more accurate initiation patterns or expression levels

The influence of insert size on MPRA-derived TSS profiles or TSS strength has not been studied before. Given that core promoter sequences (−40 to + 40 from the TSS) are typically sufficient to determine where transcription initiates (50), we hypothesized that *epi-short* TSS-MPRA inserts that did not replicate endogenous initiation profiles and had weaker evidence for TATA and Inr elements lacked important sequence features outside of the -110- to + 43-nt sequences captured by the *epi-short* plasmid pool.

To determine whether increasing the insert size of cloned sequences would lead to a more accurate transcription initiation landscape we designed a second oligonucleotide pool (*epi-long*) with longer versions of the same 250 randomly chosen promoters as the *epi-short* pool. By taking advantage of the longest commercially available oligonucleotide synthesis process (350 nt length), we designed an *epi-long* library with 303 nt of genomic sequence. Each 350 nt insert consisted of 75 nt of additional flanking genomic sequence on either side of the original *epi-short* insert sequence for 303 nt total insert length, plus 47 nt for downstream 11-mer barcodes and flanking homology arms for Gibson assembly as above. Each genomic insert sequence was barcoded 2-fold redundantly, for a total of 500 inserts.

**Figure 3.**
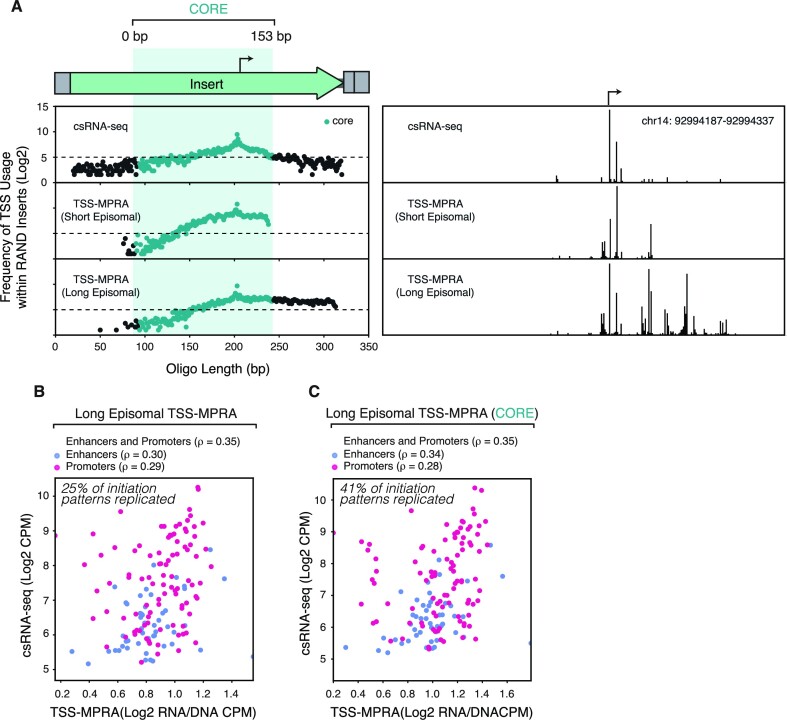
Longer inserts initiate transcription at additional non-endogenous TSSs and decrease overall TSS-MPRA transcription initiation fidelity. (**A**) Non-native TSS use in *epi-long* TSS-MPRA. Frequency of TSS usage between csRNA-seq (top), short TSS-MPRA (middle), and long TSS-MPRA (bottom). The y-axis, TSS usage frequency, is defined as the oligo position-specific cumulative normalized initiation frequencies in TSS-MPRA and csRNA-seq across all native TSS-MPRA inserts and corresponding genomic regions. At the top a schematic representation of the insert-containing oligos: overhang cloning sequence, followed by genomic DNA insert, 11-mer barcode and second overhang cloning sequence. The box and dots in blue marked ‘CORE’ are the positions covered by the 153-bp insert of the *epi-short* pool. (**B**) Increased insert length increases enhancer but not promoter transcription correlation between TSS-MPRA and csRNA-seq. Spearman's correlation of *epi-long* TSS-MPRA and csRNA-seq levels between all (purple) 250 regulatory sequences selected to cover a wide range of transcription levels and initiation patterns, or of only enhancers (blue), or only promoters (red). (**C**) Increased correlation of CORE (153-bp region) initiation frequencies within longer inserts of enhancers but not promoters. This analysis is restricted to the 153-bp region marked ‘CORE’ in (A). Color scheme as in (B).

Performing TSS-MPRA, we found that the increased insert size of the *epi-long* plasmid pool did not improve the TSS-MPRA initiation patterns of the 37% of promoter inserts that did not previously match their endogenous profiles in the *epi-short* library. Additionally, the overall fraction of promoters that recapitulated endogenous initiation patterns by WIP/SVM scoring decreased from 63% in the *epi-short* pool to 25% in the *epi-long* pool. This drop in fidelity was mostly the result of additional TSSs that were not present in the corresponding csRNA-seq data. These exogenous TSSs occurred outside and mostly downstream of the 153-bp ‘core’ insert sequence present in the *epi-short* pool (Figure 3A). Consequently, restricting the WIP analysis to the 153-bp core region of *epi-long* sequences increased the percentage of inserts with similar initiation patterns to their endogenous loci from 25% to 41%, with the majority of core initiation patterns not being affected by the genomic sequence added to the *epi-short* inserts. Decrease in initiation pattern fidelity was accompanied by a drop in correlation between transcription levels of the *epi-long* constructs and csRNA-seq (ρ = 0.35; Figure 3B), which remained the same when only quantifying initiation in the ‘core’ regions of the long inserts (Figure 3C).

When stratifying inserts by their genomic annotation we observed that with increasing insert size, the transcription levels of enhancers, but not of promoters, correlated better with endogenous transcription levels (Figures 1C, 3B). This indicates that accurate enhancer transcription (and potentially function) relies on additional sequence information encoded within longer stretches of genomic DNA than promoters, such as bidirectional initiation sites (51).

### Chromatinization by lentiviral transduction affects transcription levels of reporter constructs but not initiation patterns

**Figure 4.**
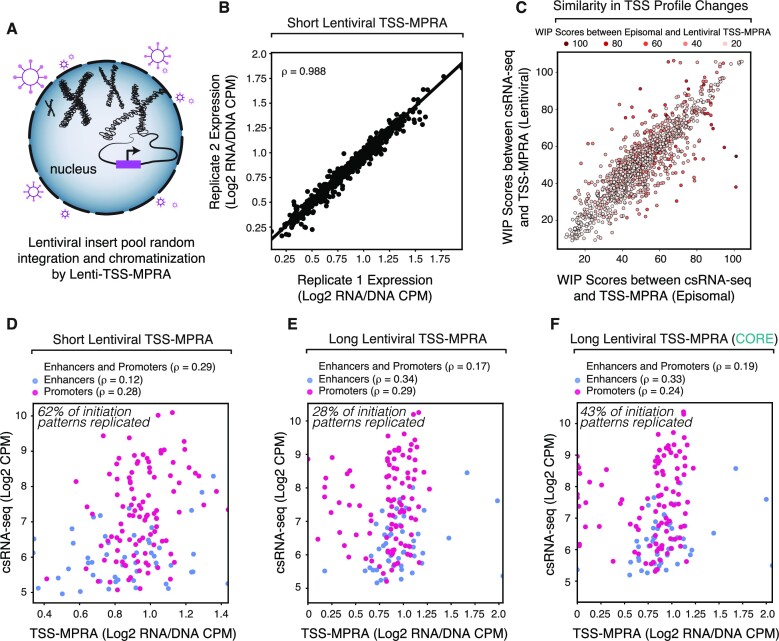
Reporter chromatinization has negligible effect on transcription initiation patterns and lowers correlation between TSS-MPRA and csRNA-seq transcription levels. (**A**) Schematic of genomic integration of a synthetic insert using lentiviral integration. (**B**) High reproducibility of Lenti-TSS-MPRA. Spearman's correlation of RNA/DNA normalized levels between all the inserts of two replicate lentiviral TSS-MPRA experiments. (**C**) High reproducibility in TSS profile changes between episomal and lentiviral TSS-MPRA experiments. WIP scores between csRNA-seq and lentiviral TSS-MPRA on the y-axis are highly correlated with the WIP scores between csRNA-seq and episomal TSS-MPRA on the x-axis. Dots are colored by how dissimilar insert TSS profiles are between lentiviral and episomal TSS-MPRA experiments. Higher WIP scores represent greater dissimilarity. (**D**) Spearman's correlation of *lenti-short* Lenti-TSS-MPRA and csRNA-seq levels between all 250 randomly selected regulatory sequences covering a wide range of transcription levels and initiation patterns (purple), only enhancers (blue), or only promoters (red). (**E**) Spearman's correlation of *lenti-long* Lenti-TSS-MPRA and csRNA-seq levels between all 250 randomly selected regulatory sequences covering a wide range of transcription levels and initiation patterns (purple), only enhancers (blue) or only promoters (red). (**F**) Spearman's correlation of *lenti-long* Lenti-TSS-MPRA and csRNA-seq levels between all 250 randomly selected regulatory sequences covering a wide range of transcription levels and initiation patterns (purple), only enhancers (blue), or only promoters (red). This analysis is restricted to the 153-bp region marked ‘CORE’ in Figure 3A.

Studies comparing transiently transfected episomal against lentivirally chromatinized enhancer reporters have suggested that lentivirally integrated reporters are more reflective of endogenous *cis*-regulatory activity (52,53). To test whether chromatinization of promoters in TSS-MPRA would similarly lead to more endogenous-like reporter transcription initiation patterns and levels, we adapted a previously published lentiviral MPRA plasmid (35) for TSS-MPRA (see Methods). We used this lenti-TSS-MPRA plasmid to clone our short and long oligo pools, generate lentivirus and integrate the pooled libraries into the genome of K562 cells in duplicate experiments (Figure 4A). Analysis of lenti-TSS-MPRA RNA and the corresponding DNA-seq libraries showed that alignment rates, fraction of active inserts, and correlation between RNA and DNA replicates of the lentiviral libraries were similar to the episomal TSS-MPRA libraries (ρ > 0.99 for both; Figure 4B, Supplementary Figure S9).

WIP analysis of lenti-TSS-MPRA data indicated that 62% of sequences in our *lenti-short* experiments mirrored their endogenous initiation patterns, which dropped to 28% in *lenti-long* libraries. These results were similar to the observations seen with episomal constructs (63% and 25% respectively), with episomal and chromatinized reporters sharing the majority of inserts that recapitulate endogenous TSS profiles (Figure 4C).

In contrast to the similarity of TSS profiles between episomal and lentivirally integrated reporters, genomic integration decreased the correlation between lenti-TSS-MPRA and endogenous transcription activity when compared to episomal experiments (*lenti-short* ρ = 0.29, *lenti-long* ρ = 0.17; Figure 4D–F). Taken together, our results suggest that while reporter chromatinization affects the overall activity of some promoters, it has little effect on reporter insert TSS profiles.

### Large (up to 950 bp) TSS-MPRA promoter inserts do not improve MPRA initiation pattern fidelity

Given that neither increasing the insert size to the maximum length possible due limitations in oligonucleotide synthesis (∼350 bp) nor lentiviral chromatinization increased the number of TSS-MPRA inserts whose initiation patterns matched their endogenous patterns, we next asked whether the previously tested DNA inserts were simply too short to contain the necessary features to fully capture some of the corresponding endogenous initiation patterns. For example, the 303-nt inserts of the *epi-* and *lenti-long* TSS-MPRA constructs only spanned from −185 nt to + 118 nt relative to the main TSS of each promoter, too short to capture the potential effects of putative nucleosome positioning sequences downstream of promoters (54,55), which are located at + 50 nt to + 200 nt from the TSS (56).

To test the degree to which even longer inserts affect TSS profile fidelity, we chose 3 representative genomic loci each for each of four categories that previously analyzed inserts fell into (12 loci in total): (i) inserts that did not match the endogenous genomic initiation profile, regardless of insert size, (ii) inserts that matched the genomic initiation profile at 153 bp but not at 303 bp length, (iii) sequences in which the core 150-bp initiation profiles differed between 153 bp and 303 bp insert size and (iv) sequences where the reporter-derived initiation profile always mirrored the endogenous initiation pattern, regardless of insert size. For each genomic locus, we PCR-amplified three inserts of increasing size (450 bp, 700 bp, 950 bp), for a total of 36 sequences and cloned them into either the pTSS-MPRA or lenti-TSS-MPRA vector.

Analysis of the TSS profiles of these inserts by TSS-MPRA in both episomal and lentivirally chromatinized settings showed that additional DNA sequence context did not improve the fidelity of MPRA-driven transcription initiation in any of the twelve cases we studied. Visualization of each TSS profile (Supplementary Figures S10–S21; see Supplementary Table S4 for locus characteristics) confirmed our previous observations that longer insert sequences give rise to more non-endogenous transcription initiation events than shorter sequences. Even after focusing on the 153-bp core of each sequence, we did not find evidence that additional DNA sequence leads to more endogenous-like TSS profiles. In fact, we rarely saw any changes in TSS profiles in the core regions with different insert sizes, as exemplified by sequences that never recapitulate endogenous initiation (Group 1; Supplementary Figures S10–S12) or sequences that always recapitulate endogenous initiation (Group 4; Supplementary Figures S19–S21), even after normalizing for potential sequencer size bias against longer transcripts (Supplementary Figures S10–S21; see tracks labeled ‘norm’; see Methods for details) (39).

Overall, our data indicates that increasing insert sizes to up to almost 1 kb did not improve the fidelity of the insert core initiation patterns relative to the initiation profiles observed at the endogenous loci.

### TSS-MPRA can be used to study the effect of motif mutations on initiation patterns and transcription levels

The function of regulatory sequences is thought to be encoded by short DNA sequence motifs that recruit transcription factors. How exactly the molecular machinery interprets the motif grammar inscribed in the genome remains poorly understood. To demonstrate the utility of TSS-MPRA in decoding regulatory grammar, we used it to analyze the effects of mutating core promoter and transcription factor motifs on the initiation patterns and transcription levels of *cis*-regulatory elements. We focused on a subset of 21 regulatory motifs enriched in K562-transcribed or accessible regulatory elements (as determined by csRNA-seq (5) and DNase-seq (57) respectively). Altogether, the *epi-short* library contained 47 control sequences that harbored various combinations of core promoter and transcription factors motifs, and 71 mutated sequences where all instances of a given motif in the control sequence were replaced with a sequence that does not match any known transcription factor motif. Each sequence was barcoded 4-fold redundantly for a total of 188 control and 284 mutated inserts.

**Figure 5.**
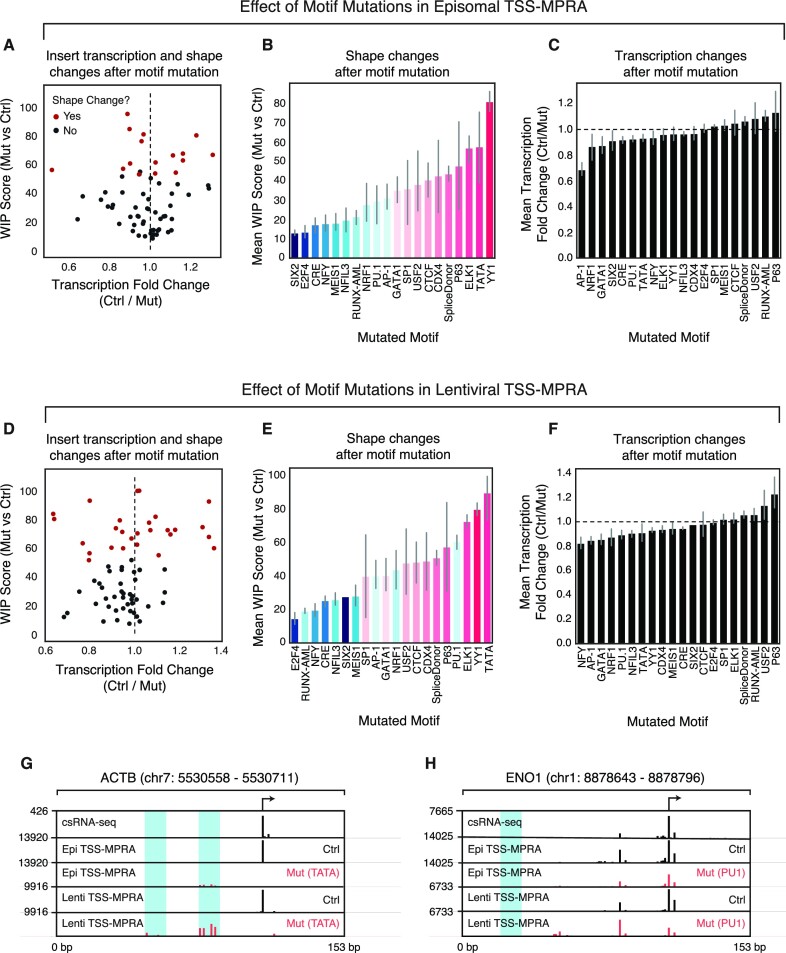
TSS-MPRA enables studying the effect of motif mutations on reporter-driven initiation patterns and transcription levels. (**A**) Tracking transcription initiation changes caused by mutations in transcription factor and core promoter element motifs in episomal plasmids. Scatterplot comparing the changes in initiation patterns (WIP score, y-axis) and transcription levels (fold change, x-axis) between control and mutated inserts in episomal plasmids. Red dots signify inserts with significantly changed TSS shapes after mutation. (**B**) TSS shape changes after motif mutation in episomal constructs. The y-axis represents the mean WIP score between all inserts (and their barcode replicates) containing a particular motif and the corresponding insert with the mutated motif. Colors correspond to motif identities. (**C**) Transcription level changes associated with motif mutation in episomal constructs. The y-axis represents the mean fold transcription change of the inserts (and their barcode replicates) containing a particular wild-type or mutated motif (x-axis). (**D**) Tracking transcription initiation changes caused by transcription factor and core promoter element motif mutations after lentiviral integration into the genome. Scatterplot comparing the changes in initiation patterns (WIP score, y-axis) and transcription levels (fold change, x-axis) between control and mutated inserts in lentiviral plasmids. Red dots signify inserts with significantly changed TSS shapes after mutation. (**E**) TSS shape changes after motif mutation in lentiviral constructs. The y-axis represents the mean WIP score between all inserts (and their barcode replicates) containing a particular motif and the corresponding insert with the mutated motif. Colors correspond to motif identities. (**F**) Transcription level changes associated with motif mutation in lentiviral constructs. The y-axis represents the mean fold transcription change of the inserts (and their barcode replicates) containing a particular wild-type or mutated motif (x-axis). (**G**) Example track for the ACTB promoter, showing csRNA-seq (top), TSS-MPRA, and Lenti-TSS-MPRA output without (black) and with (red) TATA-box motif mutation. Blue highlights indicate the positions where motifs were replaced by a constant sequence with no known transcription factor motif. (**H**) Example track for the ENO1 promoter, showing csRNA-seq (top), TSS-MPRA, and Lenti-TSS-MPRA output before (black) and after (red) PU.1 motif mutation. Blue highlights indicate the positions where motifs were replaced.

Despite the small overall number of sequences studied, DNA motif mutations induced clearly visible and significant initiation pattern changes in 25% of regulatory elements when mutating all instances of the respective DNA motif (Figure 5A). Most of these changes were the result of mutating a small number of motifs (Elk1, YY1, TATA-box; Figure 5B), while mutations in other motifs caused either minimal or varied effects. In contrast, we found that motif mutations only modestly affected insert transcription levels, with mutations in AP-1 and p63 motifs having the largest impacts (Figure 5C).

We observed that reporter chromatinization increased the fraction of inserts with significantly transformed TSS profiles from 25% to 40% (Figure 5D). Interestingly, we found that mutations in motifs for the Ets factor PU.1 led to larger WIP scores (indicative of more significant changes in initiation pattern) only when the reporter library was properly chromatinized (Figure 5E, H), consistent with its known role in chromatin opening (58,59). However, similar to the results in our episomal experiments, motif mutations had a minimal effect on transcription levels of chromatinized inserts (Figure 5F). The large impact of TATA-box mutations on initiation patterns in both episomal and chromatinized contexts corroborates its well-known role in positioning the pre-initiation complex and RNAPII to initiate transcription (60) (Figure 5G). These results highlight the value of TSS-MPRA to investigate *cis*-regulatory grammar, to gain an understanding of how transcription factor binding sites contribute to both activity levels and initiation site usage of regulatory sequences.

### TSS-MPRA can be used to study the effect of single-nucleotide polymorphisms on initiation patterns and transcription levels

Given that greater than 80% of disease-associated genetic variants reside within non-coding regions with likely gene-regulatory activity, it is essential for their interpretation to better understand how these SNPs impact regulatory function (61). Recent studies have used MPRAs to evaluate how SNPs in putative enhancers affect transcription. To test the degree to which TSS-MPRA can be used to study the influence of SNPs on transcription initiation patterns and levels, we analyzed the effect of 45 SNPs from the GWAS catalog that overlapped regulatory elements transcribed in K562 cells on the transcription initiation landscape of episomal and chromatinized reporters.

**Figure 6.**
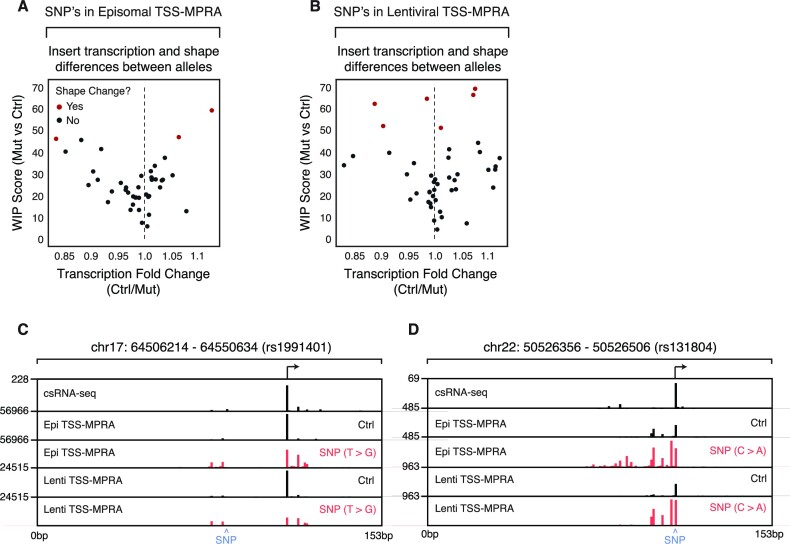
Assessing the effects of single nucleotide polymorphisms on reporter-driven initiation patterns and transcription levels. (**A**) Allele-specific transcription initiation differences caused by known GWAS SNPs in episomal plasmids. Scatterplot comparing the changes in initiation patterns (y-axis) and transcription fold change (x-axis) between control and variant inserts in episomal constructs. Red dots signify inserts that had significant changes to their TSS shapes. (**B**) Allele-specific transcription initiation differences caused by known GWAS SNPs in lentiviral constructs. Scatterplot comparing the changes in initiation patterns (y-axis) and transcription fold change (x-axis) between control and variant inserts in lentivirally integrated constructs. Red dots signify inserts that had significant changes to their TSS shapes. (**C**) SNP rs1991401 is associated with TSS shape changes. Track showing csRNA-seq (top), TSS-MPRA, and Lenti-TSS-MPRA output of the T (black) and G (red) allele. A blue ‘^’ symbol indicates the location of the SNP. (**D**) TSS shape differences associated with SNP rs131804. Track showing csRNA-seq (top), TSS-MPRA, and Lenti-TSS-MPRA output of the C (black) and A (red) allele. A blue ‘^’ symbol marks the SNP location.

Three of the forty-five SNPs tested (∼7%) were associated with dramatic differences in TSS profile in episomal TSS-MPRA experiments (Figure 6A). Reporter chromatinization increased the number of SNPs that led to significantly different initiation patterns to 6 (∼14%) (Figure 6B). As an example, the SNP rs1991401, which is associated with myopia, asthma and blood traits (62–65), affects the TATA-box in the promoter of the *DDX5* gene. TSS-MPRA results of inserts harboring the rs1991401 variant show a pronounced shift to a dispersed initiation phenotype that is consistent with the loss of TATA-box function (Figure 6C), with little effect on transcript levels. On the other hand, SNP rs131804, which is also associated with blood traits, causes a mutation 2 bp upstream of the primary TSS in the *SCO2* gene, leading to a substantial change in initiation site usage and higher overall levels of TSS-MPRA activity (Figure 6D).

Our results demonstrate that TSS-aware MPRAs can identify variants associated with extreme changes in initiation patterns, which provides additional context and insight into their potential regulatory impact that may be missed when using conventional MPRA approaches that only measure transcription levels.

## DISCUSSION

The principle of quantifying reporter transcripts by sequencing to determine the effect of sequence changes in the promoter regions has enabled massively parallel analysis of sequence features in promoters and enhancers (18,20,21). The first and most-widely used implementation of this approach distinguishes reporter transcripts based on unique barcodes at their 3′ ends. One drawback of 3′ barcoding when studying promoter elements is that the resulting data carries no information about where transcription initiates, which is an important promoter feature that directly relates to regulatory mechanisms (7,8,17,25). More recently, 5′ end-aware methods have been developed, which sequence reporter transcript 5′ ends to provide data on both transcription initiation location as well as transcription levels (6,25,26).

With MPRAs increasingly being used to study the functional consequences of genetic variation and the fact that transcription initiation patterns are linked to mechanisms of transcription regulation (7,8,17,25), it is essential to know how well promoters in an MPRA replicate their initiation patterns in the genome. The only comparison of this kind to date (6) suggested that MPRA and endogenous initiation patterns agree. However, as this analysis was done by comparing averaged initiation signals across many promoters, and in *drosophila*, where TSS profiles may (66) or may not be largely focused (34), the question whether the TSS patterns of individual human promoters in the MPRA match their endogenous counterparts remained unanswered.

To address this issue, we used TSS-MPRA to compare individual MPRA-derived and endogenous TSS profiles of randomly chosen human promoters. For sensitive quantification of TSS profile differences, we additionally developed a new computational approach (WIP score) that outperforms the dissimilarity metric most commonly used for this purpose, EMD (48) (Supplementary Figures S5–S7) on experimental data. Using WIP scores to quantify TSS-MPRA and endogenous profile differences, we found that 37% of MPRA inserts did not recapitulate endogenous TSS profiles when analyzing short 153 bp promoter inserts in episomal reporters.

While episomal reporter plasmids offer some level of chromatinization (67), integrating reporters into the genome via lentiviral transduction is thought to provide a more native chromatin state and has been shown to affect enhancer activity in the context of an MPRA (52). To our surprise, lentiviral integration of TSS-MPRA constructs into the genome did not change TSS fidelity of the MPRA (38% of endogenous profiles not recapitulated), with TSS patterns in the lentiviral MPRA being very similar to the episomal TSS profiles (Figure 4C). In contrast, correlation between relative insert and endogenous transcription levels (ρ_Episomal_ = 0.43, Figure 1C) was indeed affected by lentiviral chromatinization (ρ_Lenti_ = 0.29, Figure 4D), similar to the effect observed in lentivirally integrated enhancer MPRAs (52).

Another parameter that has not been characterized for promoter MPRAs is the effect of insert size. In enhancer MPRAs, insert size has been shown to affect enhancer activity (53), but as the activity of the corresponding enhancers in their native environment is unknown, it is difficult to draw meaningful conclusions from this finding. While the same applies to promoter MPRA transcription levels, endogenous TSS profiles can serve as ground truth to determine how insert size affects fidelity of MPRA TSS profiles. Our initial results comparing 303-bp and 153-bp inserts centered on the same endogenous TSS indicated that while the shorter libraries recapitulated the majority of endogenous TSS profiles (63%), the longer inserts had lower fidelity (25% recapitulated overall, 41% when only assessing the core 153-bp region of the long inserts). Testing the effect of even longer inserts of up to 950 bp centered on the TSS on transcription initiation profiles, we found that increasing the amount of sequence context around the TSS in the MPRA did not increase fidelity of the resulting TSS profiles. On the contrary, for inserts with TSS profiles that diverged from the endogenous profile or where the TSS pattern differed between 153 bp and 303 bp inserts, further increasing the insert length to up to 950 bp further increased the dissimilarity of the overall pattern (Supplementary Figures S10–S21). This was largely due to additional initiation sites mostly located downstream of the original TSS, rather than changes to the core initiation pattern.

The divergent and size-associated changes in episomal TSS-MPRA TSS patterns were mirrored in lentivirally chromatinized longer-insert reporter constructs. Possible causes for this phenomenon may include the lack of DNA methylation of the reporter gene in reporter constructs, which normally suppresses spurious initiation in gene bodies of endogenous genes (68) and a lack of proper chromatinization and chromatin context caused by the lentiviral MPRA backbone that harbors insulators to dampen effects from random integration into chromatin (52). Another reason for poor TSS fidelity could be the absence of differentiation-associated chromatin changes in relatively transient experiments in cell lines: the studies where (large) transgenes and enhancer reporters exhibit high transcriptional and epigenetic fidelity were done in pluripotent stem cells (69–72) and in whole animals (73–76), where the transgenic DNA undergoes differentiation-associated activity progressions that mirror those of the endogenous sequence (77). Thus, while additional sequence information surrounding the promoter might be necessary to help drive the correct TSS profile, it may only be doing so during cellular differentiation.

Overall, our results suggest that in the context of MPRAs, factors other than DNA sequence can affect reporter fidelity of transcription initiation patterns. Further work will be necessary to uncover the determinants of the differences in TSS patterns between reporters and endogenous loci.

Our data shows that it is essential to assess promoter TSS pattern fidelity in the MPRA before embarking on mechanistic studies. To this end, we have demonstrated how a TSS-aware MPRA and single-nucleotide resolution dissimilarity scoring can be used to select promoters for further study whose TSS patterns in the MPRA replicate the endogenous TSS profile, and how TSS-MPRA can be used to characterize the effects of promoters promoter SNP on transcription initiation. Further, we anticipate that our simplified TSS-MPRA protocol with its 10–20-fold lower input requirements compared to STAP-seq (7,8) together with the lentiviral reporter vector we adapted from lentiMPRA (35) will enable 5′-aware MPRA in cells that are not easily electroporated with DNA, and will open the door for 5′-aware MPRAs *in vivo*.

## Supplementary Material

gkad562_Supplemental_Files

## Data Availability

All of the code used to analyze and compare TSS-MPRA and csRNA-seq data in this manuscript is available on GitHub (https://github.com/c-guzman/wishbone, permanent doi: https://doi.org/10.5281/zenodo.8049238).

## Notes

Present address: Yixin Zhu, Department of Quantitative and Computational Biology, University of Southern California, Los Angeles, CA 90089, USA.

Present address: Sascha Duttke, School of Molecular Biosciences, College of Veterinary Medicine, Washington State University, Pullman, WA 99164, USA.
