# Supplementary material for: Combining TSS-MPRA and sensitive TSS profile dissimilarity scoring to study the sequence determinants of transcription initiation
Source: Nucleic Acids Res. 2023 Jul 5;51(15):e80. doi: 10.1093/nar/gkad562 (PMC10450201; doi:10.1093/nar/gkad562)
Supplement: gkad562_Supplemental_Files [file gkad562_supplemental_files.zip › SupplementaryFiles.pdf]

Supplementary Figure S1

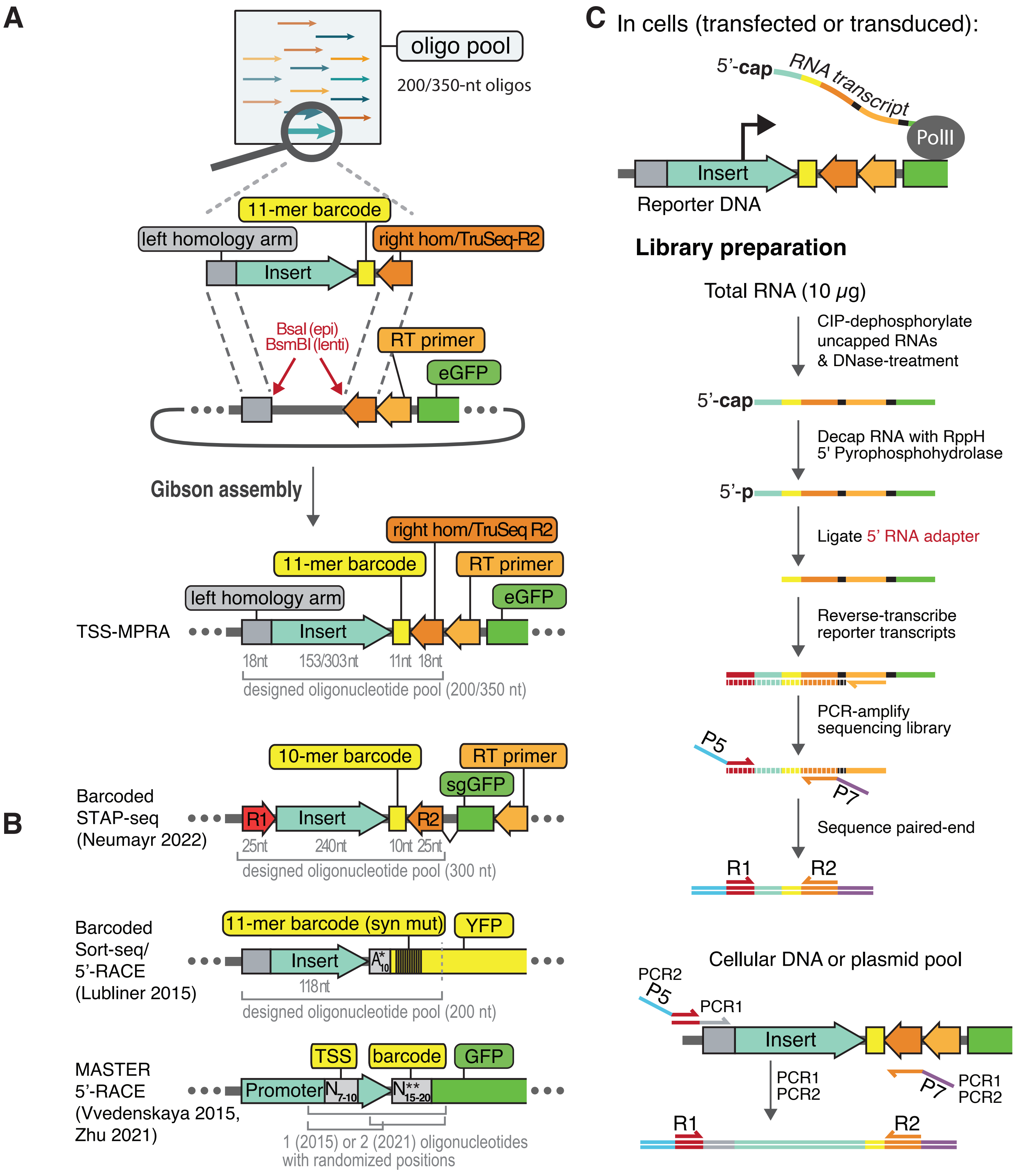

Supplementary Figure S1: Schematic for TSS-MPRA insert cloning site plasmid design and library preparation procedure. TSS-MPRA (**A**) Cloning schema and insert, barcode and primer arrangement. (**B**) Sequence features of alternative 5' MPRAs. *constant A-rich 10-mer sequence (5'-TAAATAAAAA-3') for efficient YFP translation; **N_{15} or "N_{20}": (5'-N_4AN_4CN_4GN_4TN_4-3'). (**C**) TSS-MPRA 5' RNA-seq library procedure. Primer landing sites are represented by colored and labeled full arrows or lines, primers are represented by half-arrows, cDNA is a hatched line. **cap**: methyl-guanosine triphosphate, **p**: monophosphate, P5&P7: Illumina flow cell-compatible primers, R1&R2: Illumina TruSeq Read 1 and Read 2 sequencing primers.

Supplementary Figure S2

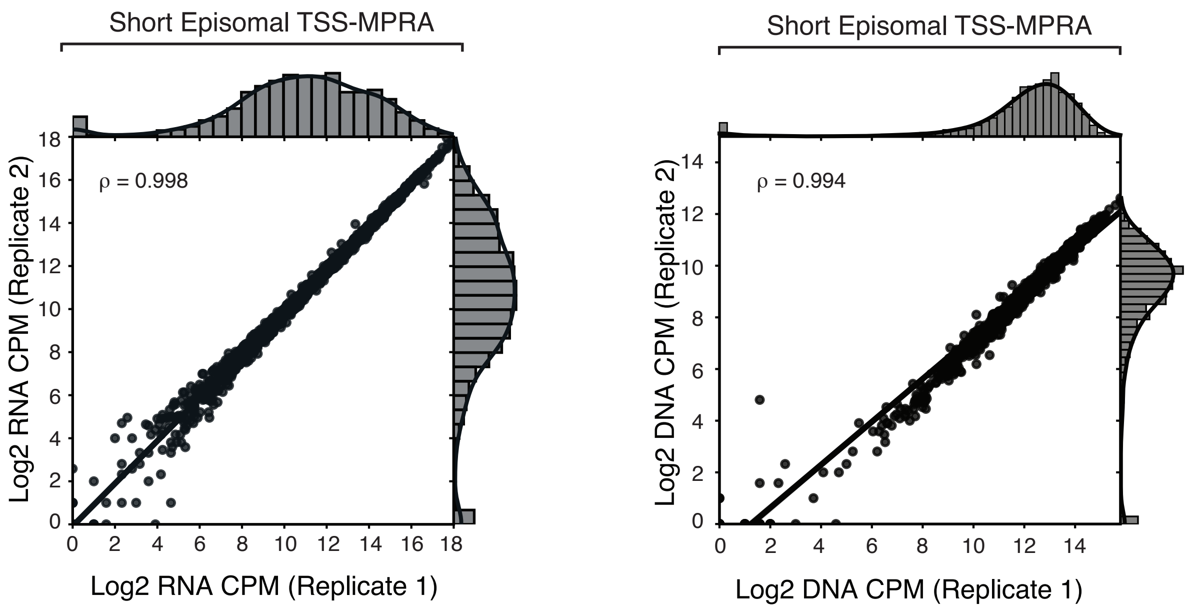

Supplementary Figure S2: Spearman correlation of RNA and DNA tag counts between two biological episomal TSS-MPRA replicates

Correlation of RNA and DNA reads normalized to counts-per-million (Log2) between all the inserts of two episomal TSS-MPRA replicates .

Supplementary Figure S3

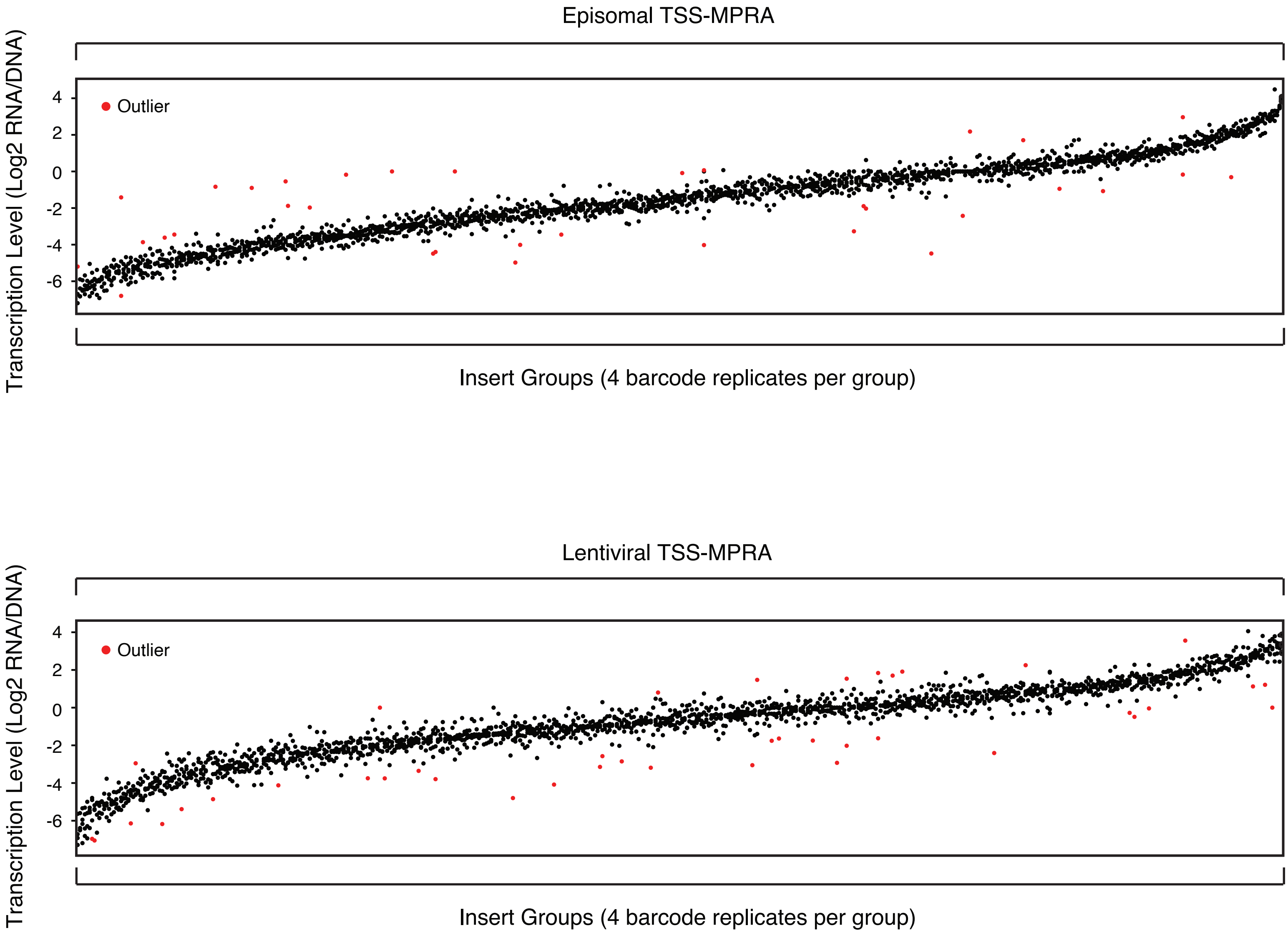

Supplementary Figure S3: Barcodes have minimal impact on TSS-MPRA output.

Scatterplot of DNA-normalized transcription levels (y-axis) per 'insert group', ordered along the x-axis by increasing median value of the DNA-normalized tag counts of each group of 4 replicate inserts with identical insert sequence but different barcodes. Red dots indicate inserts that have transcription levels at least 3 standard deviations from the mean of their insert group (34 for epi-short and 41 for lenti-short).

Supplementary Figure S4

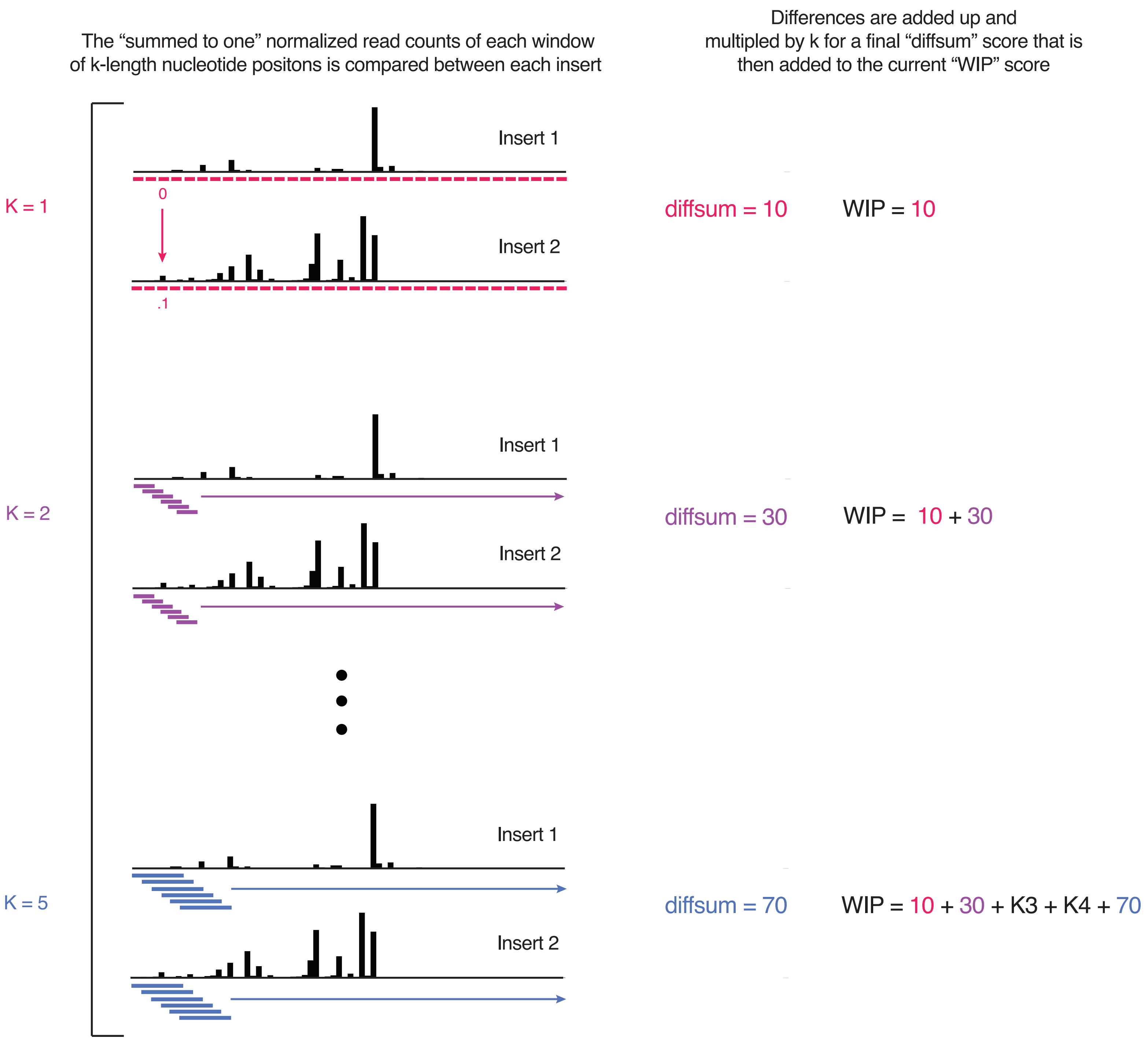

Supplementary Figure S4: Schematic of WIP score calculation.

WIP scores calculate the dissimilarity between two TSS distributions that can be represented as arrays of the same length. TSS frequencies within each insert are normalized so that they sum to one (# of reads mapped at a certain bp / total reads mapped to insert). We then apply a k-length sliding window approach where TSS frequencies from each window from one insert is compared against the same window in another insert. The absolute difference between normalized TSS frequencies at each window is added up and multiplied by k (called diffsum). This sliding window approach and diffsum score calculation is repeated up to a k of 5. Each k-diff score is added at the end to create a final score that is equivalent to the WIP score.

Supplementary Figure S5

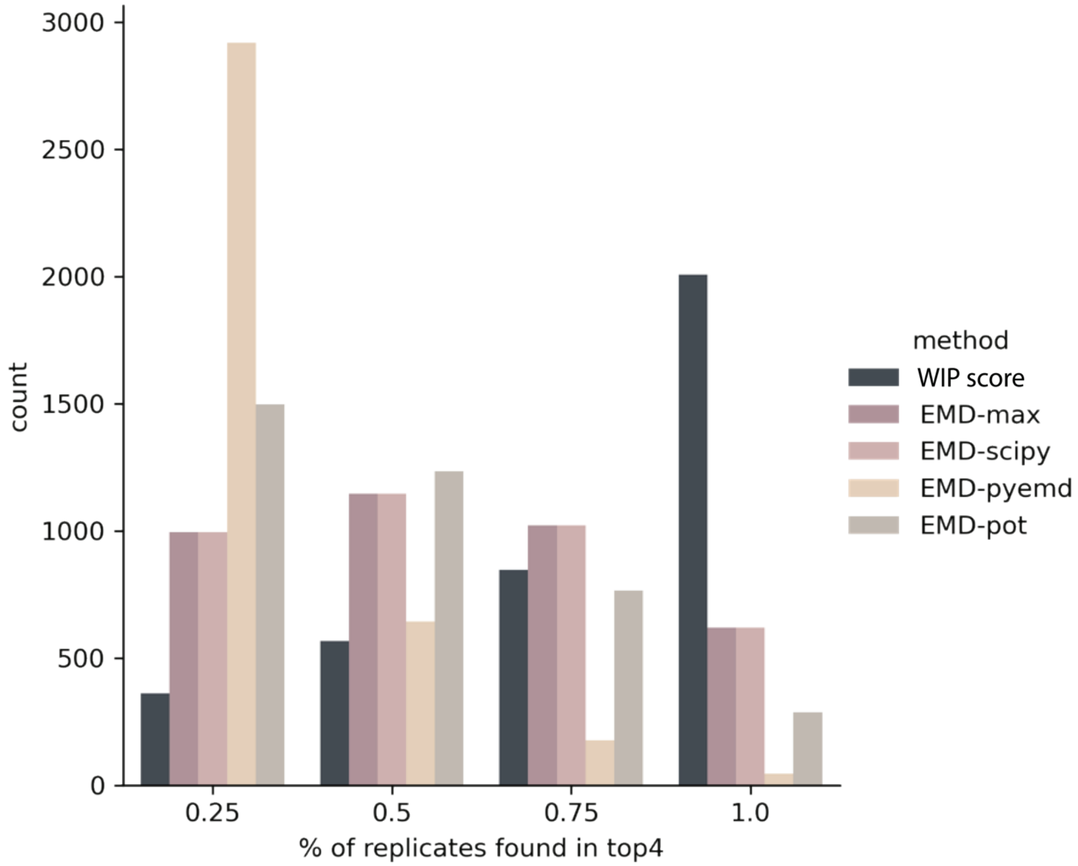

Supplementary Figure S5: WIP score more reliably identifies an insert's barcode replicates than EMD

Countplot of the percentage of an insert's barcode replicates that each method identifies being in the top four inserts with the lowest WIP scores (y-axis) across two biological replicates. The WIP score for each insert is calculated against every other insert in the pool and the number of times it's barcode replicates are found in the four lowest WIP scores is divided by 4 to give the % of replicates found in top4. Four different methods were used to calculate EMD scores: I) EMD-max is a custom in-house script, II) EMD-scipy is the EMD implementation available via SciPy, III) EMD-pyemd is the EMD implementation available via PyEMD, and IV) EMD-pot is the EMD implementation available via the Python Optimal Transport package.

Supplementary Figure S6

Celeb_HBQ_mutMotif_CTCF_Zf_BCrep2: WIP did better than EMD

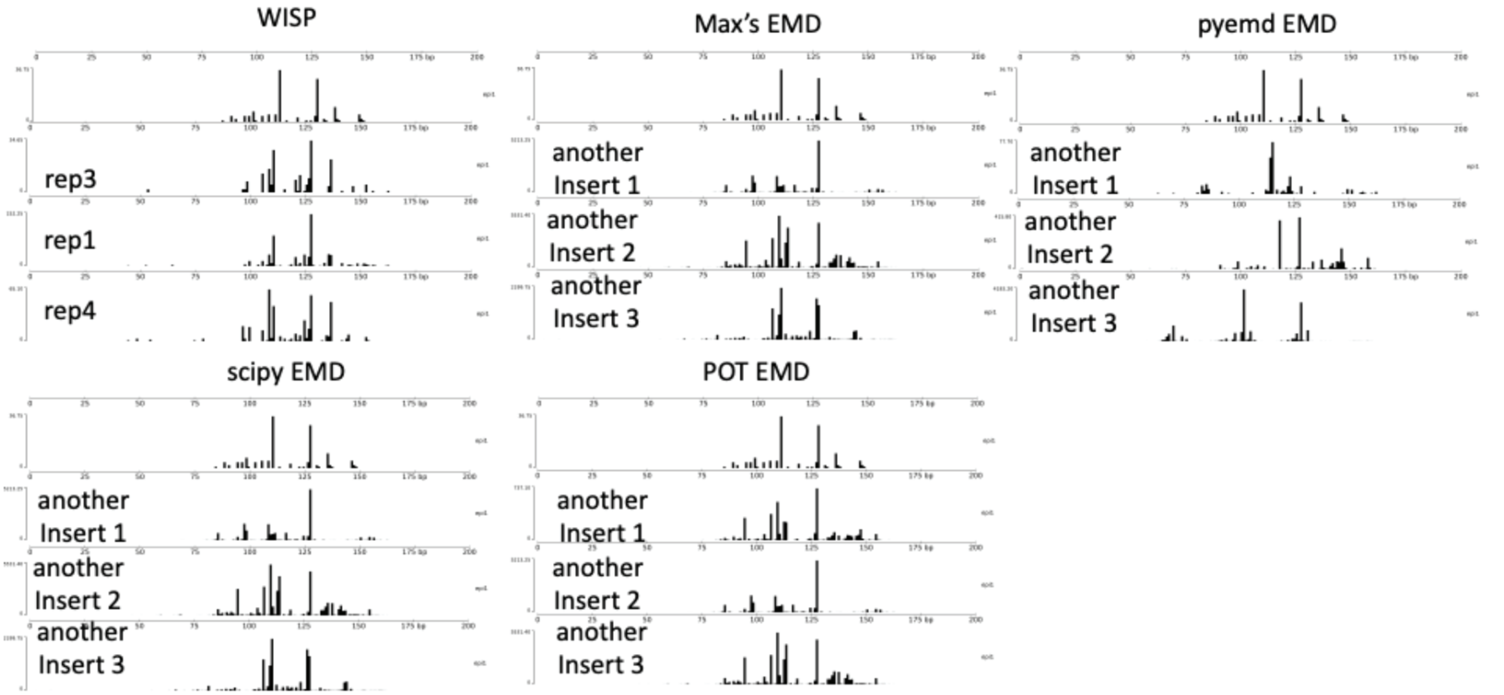

Celeb_ACTB_mutMotif_NFY_CCAAT_BCrep2: WIP did better than EMD

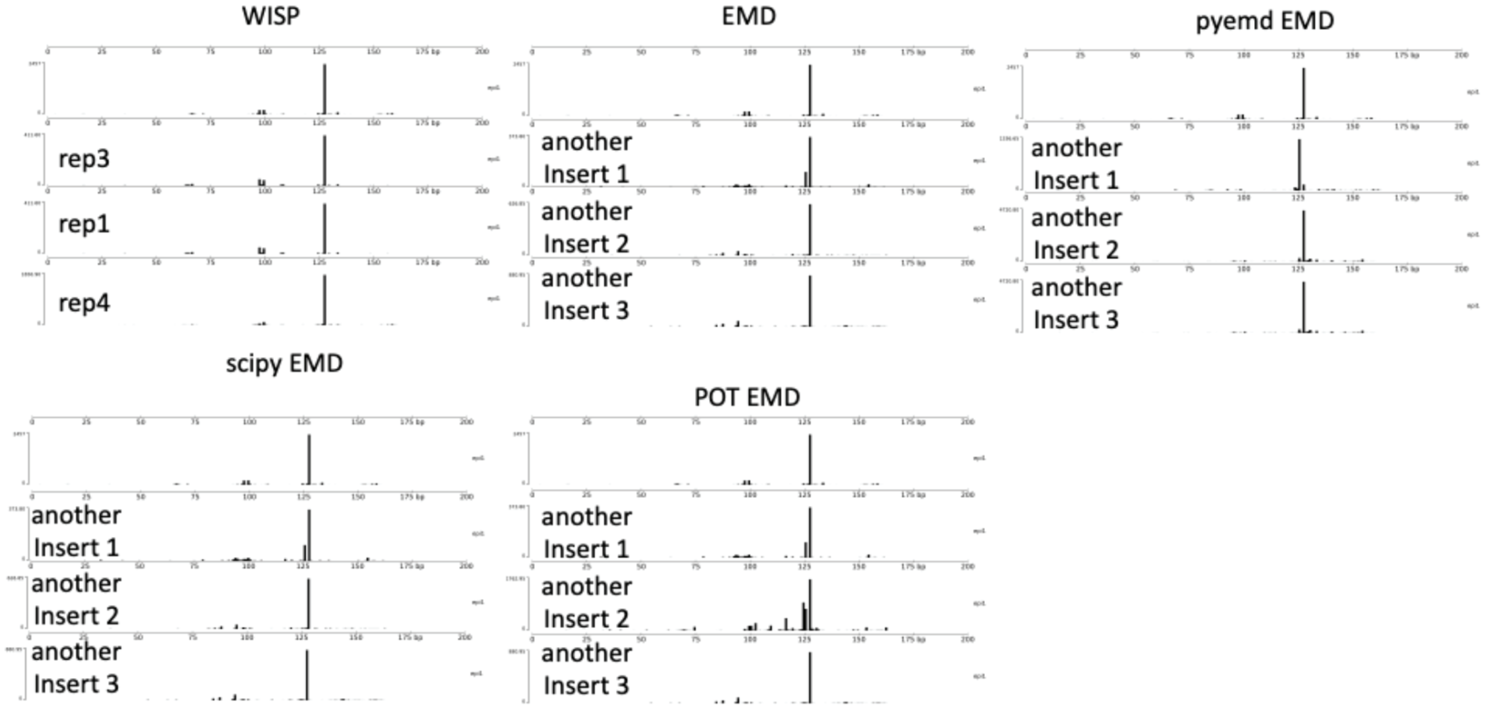

Supplementary Figure S6: Two examples where WIP scores identify more similar TSS profiles than EMD

Tracks for two different inserts where WIP scoring lead to the identification of more similiar TSS profiles than EMD. Each track represents the top 3 inserts that are calculated as most similar according to their metric.

Supplementary Figure S7

Celeb_ENO1_mutMotif_RUNX-AML_Runt_BCrep3: WIP did better than EMD

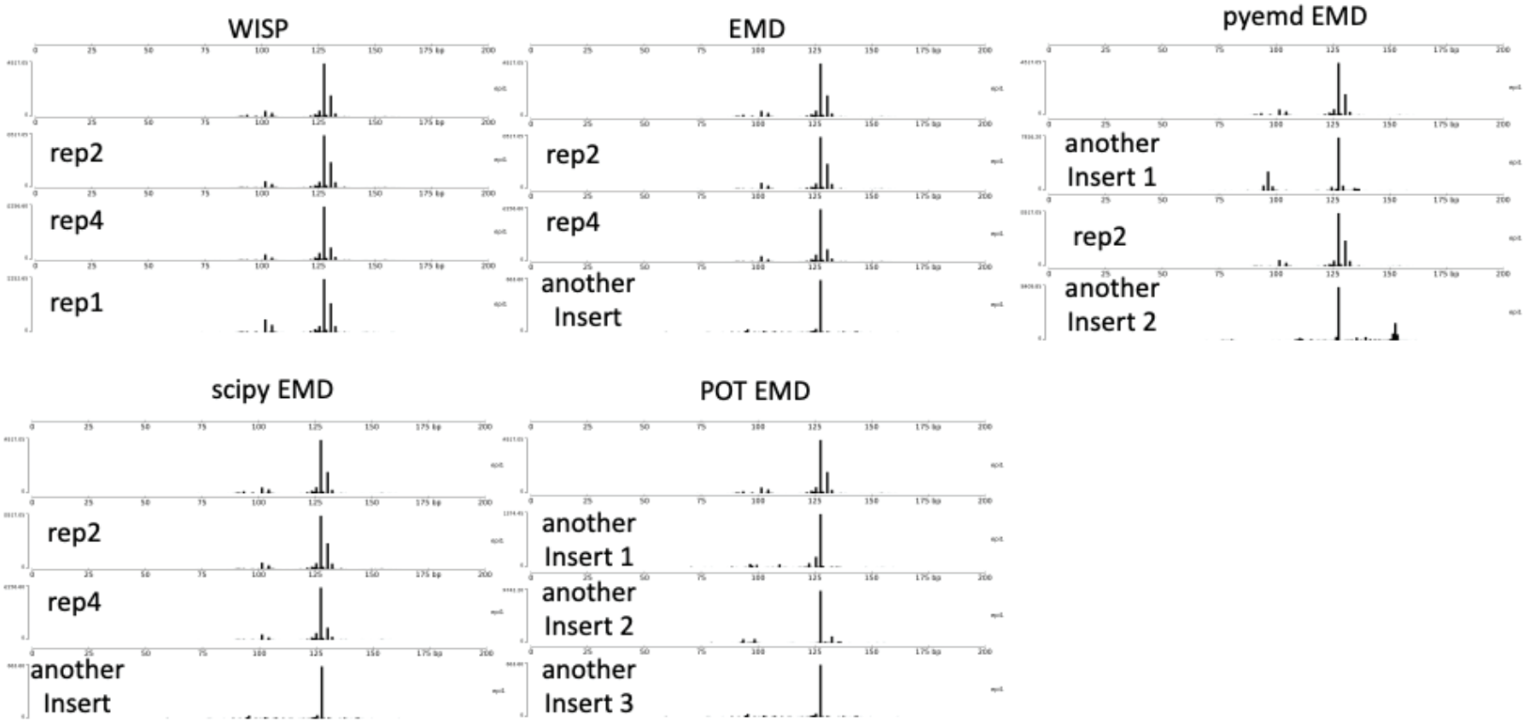

Celeb_HBE1-promoter_mutMotif_NFY_CCAAT_BCrep2: EMD did better than WIP

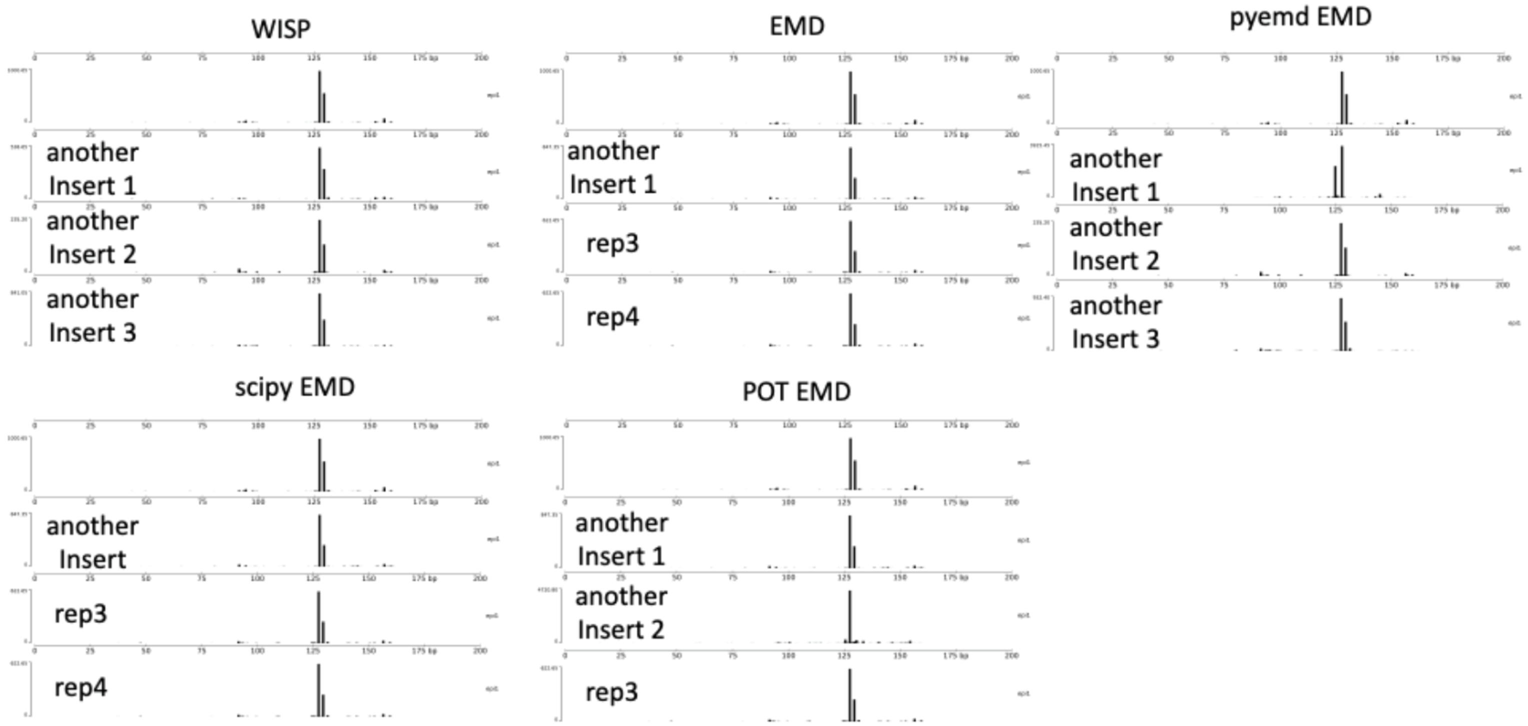

Supplementary Figure S7: Examples of inserts where EMD or WIP do better at identifying barcode replicates

Tracks for two different inserts where either WIP scoring or EMD lead to the identification of more similiar TSS profiles.
Top insert track is an example where WIP scoring finds an insert's barcode replicates amongst it's top 4 most similar matches.
Bottom insert track is an example where EMD finds more an insert's barcode replicates amongst it's top 4 most similar matches.
Each track represents the top 3 inserts that are calculated as most similar according to their metric.

Supplementary Figure S8

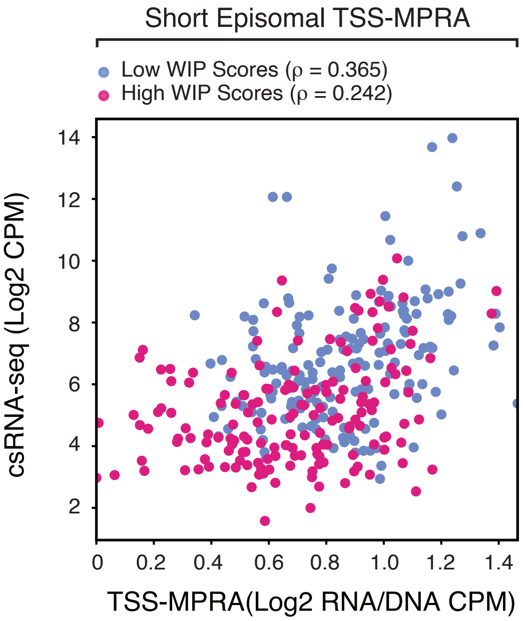

Supplementary Figure S8: Divergent initiation patterns indicate poorer correlation with endogenous transcription initiation.

Spearman's correlation of epi-short TSS-MPRA and csRNA-seq levels between all randomly selected regulatory sequences covering a wide range of transcription levels and initiation patterns and motif/SNP control sequences that greatly mirror endogenous TSS shapes (blue), or poorly mirror endogenous TSS shapes (red).

Supplementary Figure S9

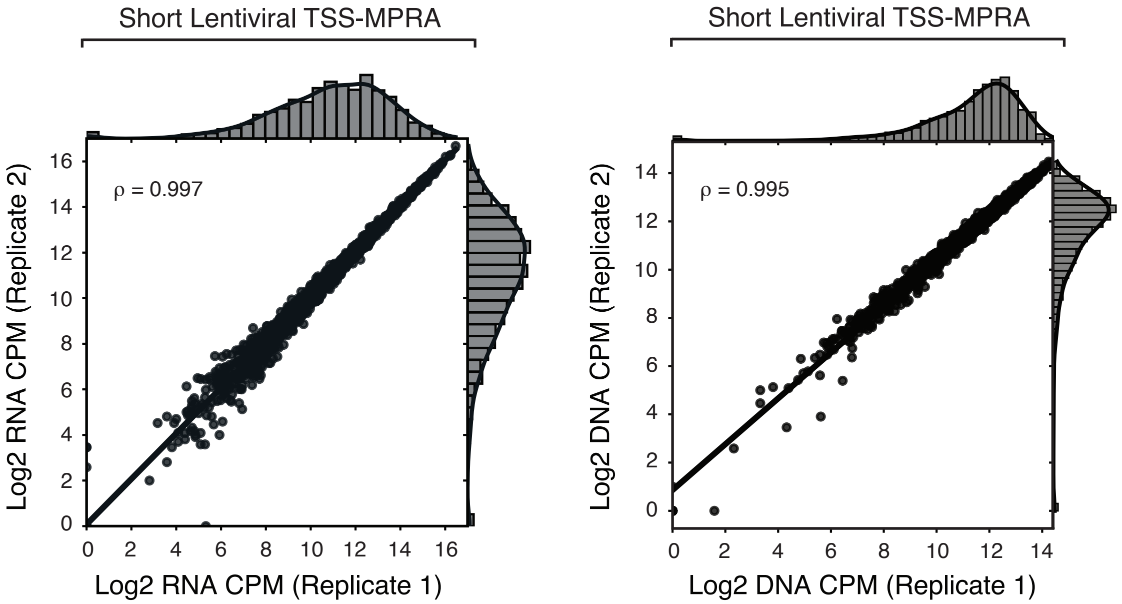

Supplementary Figure S9: Spearman correlation of RNA and DNA tag counts between two biological lentiviral TSS-MPRA replicates

Correlation of RNA and DNA reads normalized to counts-per-million (Log2) between all the inserts of two lentiviral TSS-MPRA replicates .

Supplementary Figure S10

Sequence 1

chr3119677313-119677463

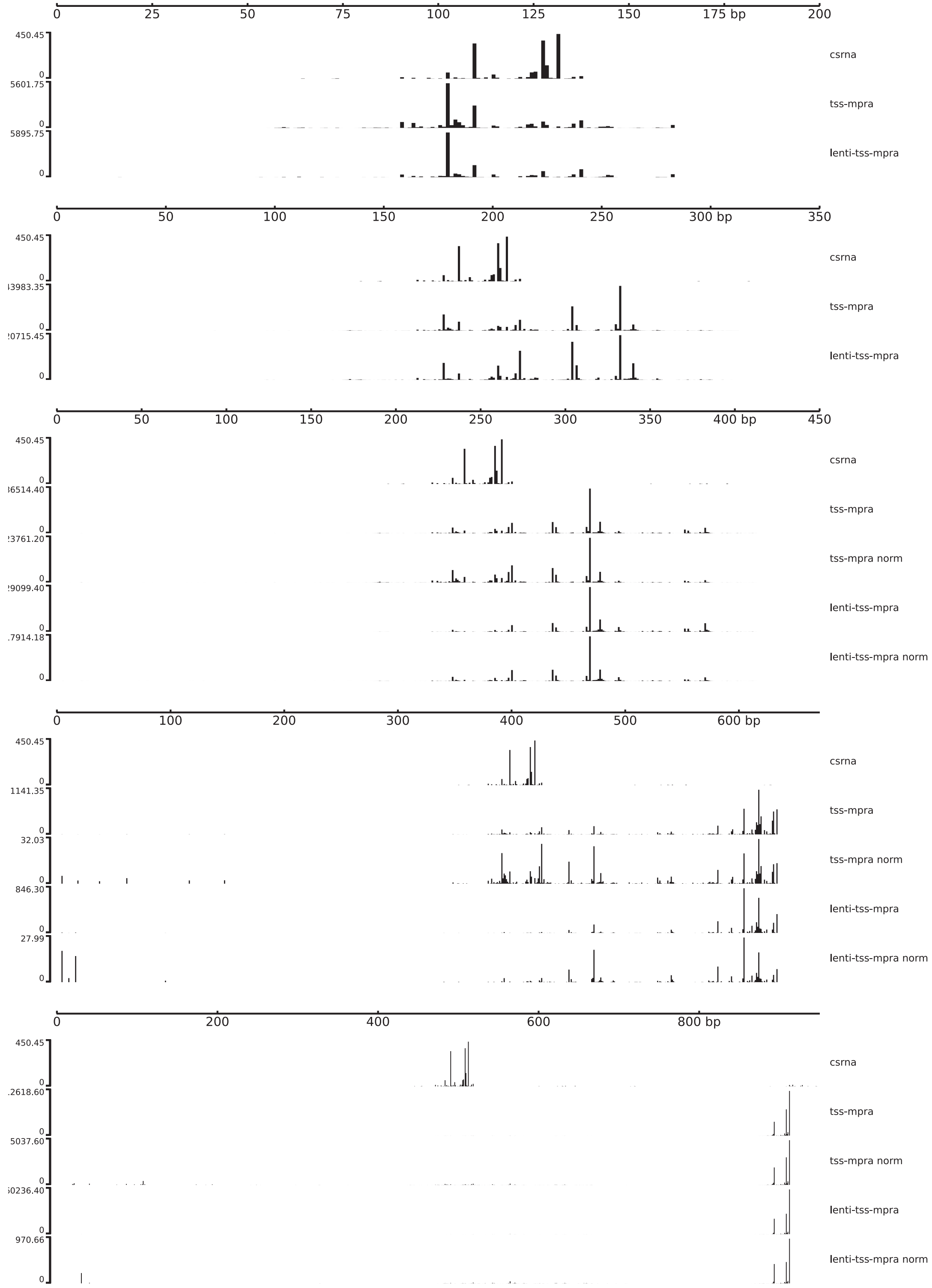

Supplementary Figure S10. TSS profiles across four different insert lengths.

RNA read counts at each bp of an insert across four different insert lengths (200bp, 350bp, 450bp, 700bp, and 950bp). The top track in each graph represents the endogenous TSS profile (csRNA-seq) while the tss-mpra and lenti-tss-mpra labeled tracks represent the TSS profiles from TSS-MPRA and Lenti-TSS-MPRA experiments respectively. Tracks labeled with ‘norm’ have been normalized for sequencing size bias.

Supplementary Figure S11

Sequence 2

chr6:33425581-33425731

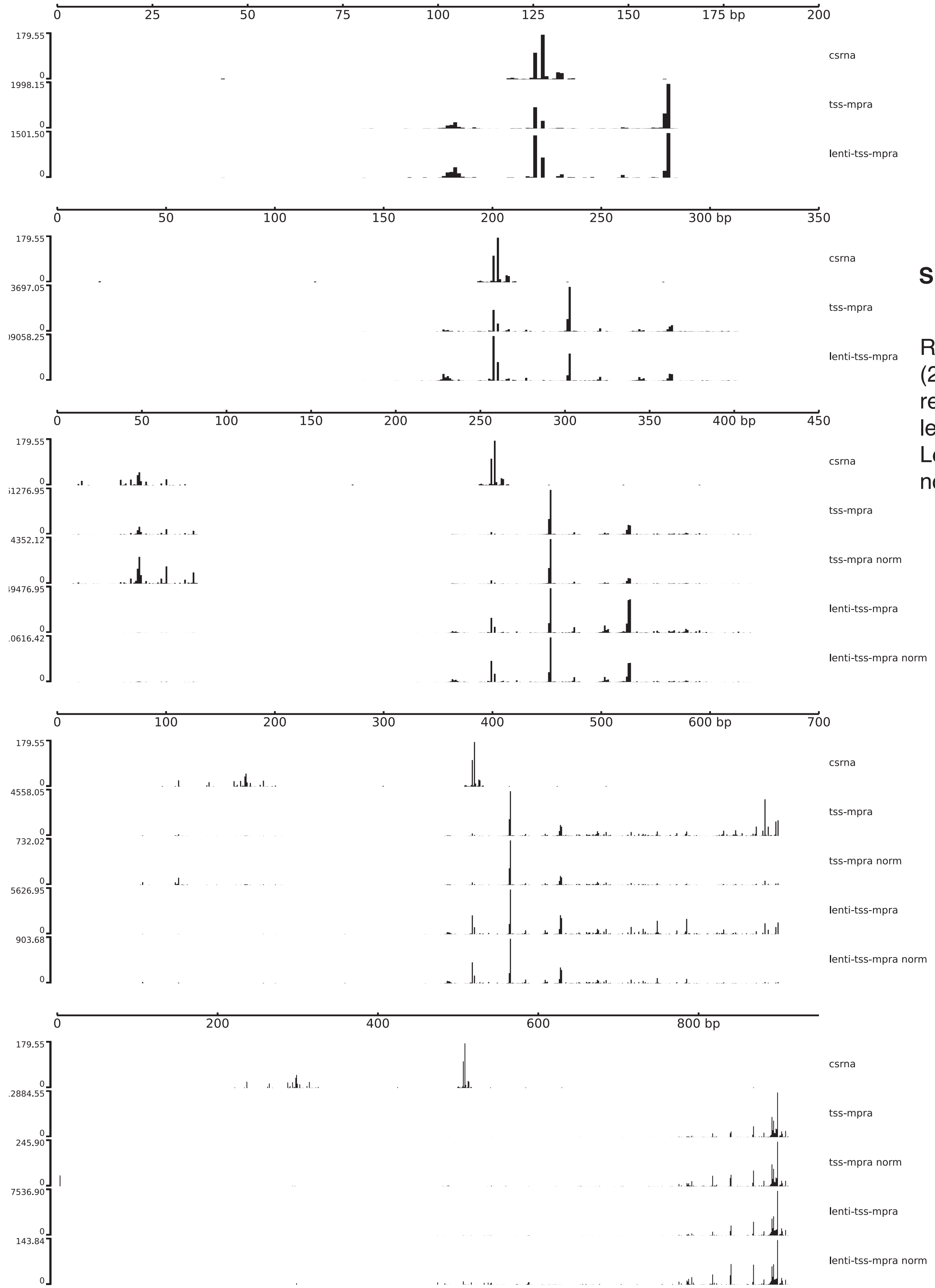

Supplementary Figure S11. TSS profiles across four different insert lengths.

RNA read counts at each bp of an insert across four different insert lengths (200bp, 350bp, 450bp, 700bp, and 950bp). The top track in each graph represents the endogenous TSS profile (csRNA-seq) while the tss-mpra and lenti-tss-mpra labeled tracks represent the TSS profiles from TSS-MPRA and Lenti-TSS-MPRA experiments respectively. Tracks labeled with ‘norm’ have been normalized for sequencing size bias.

Supplementary Figure S12

Sequence 3

chr6:31703401-31703551

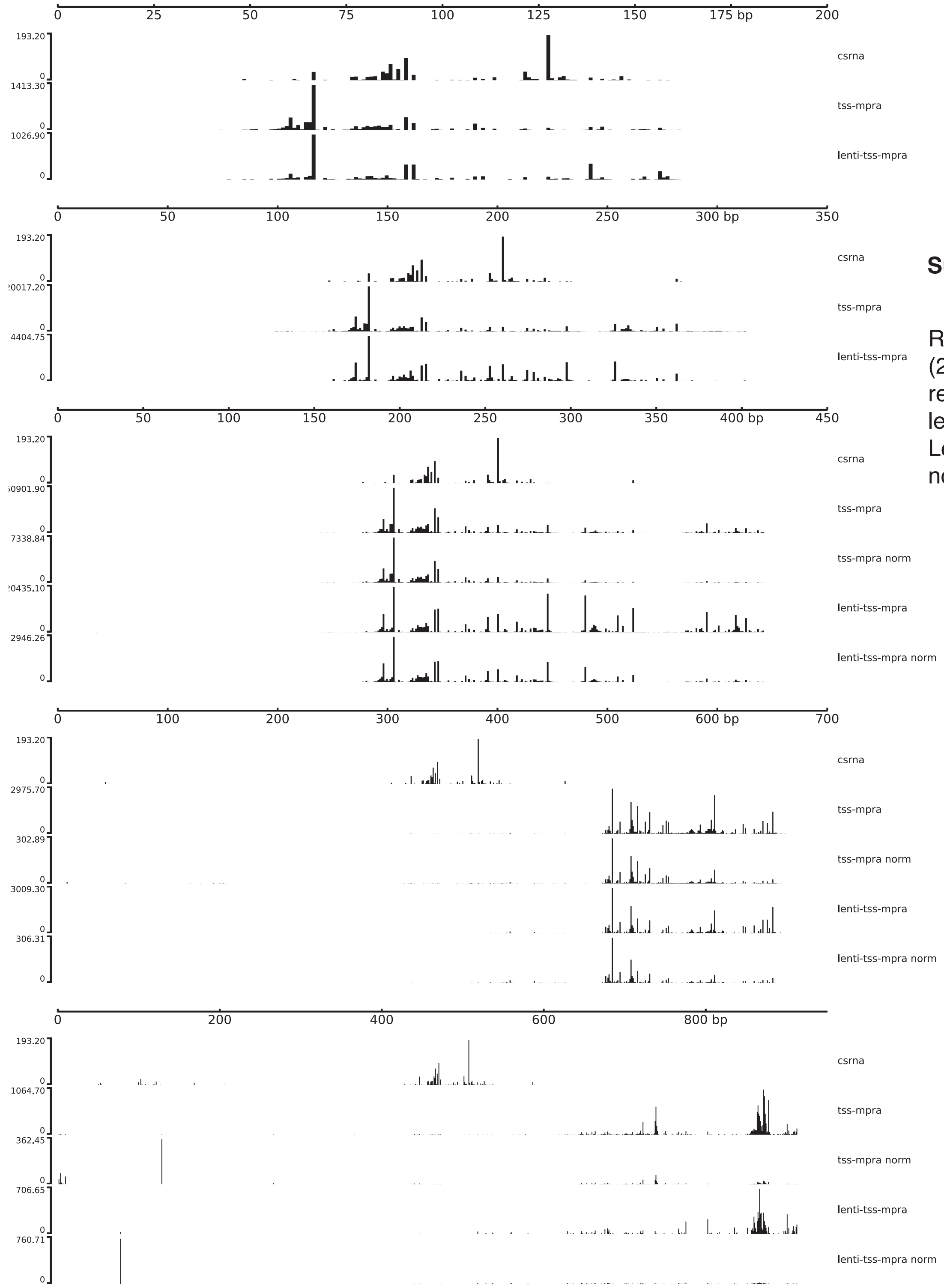

Supplementary Figure S12. TSS profiles across four different insert lengths.

RNA read counts at each bp of an insert across four different insert lengths (200bp, 350bp, 450bp, 700bp, and 950bp). The top track in each graph represents the endogenous TSS profile (csRNA-seq) while the tss-mpra and lenti-tss-mpra labeled tracks represent the TSS profiles from TSS-MPRA and Lenti-TSS-MPRA experiments respectively. Tracks labeled with ‘norm’ have been normalized for sequencing size bias.

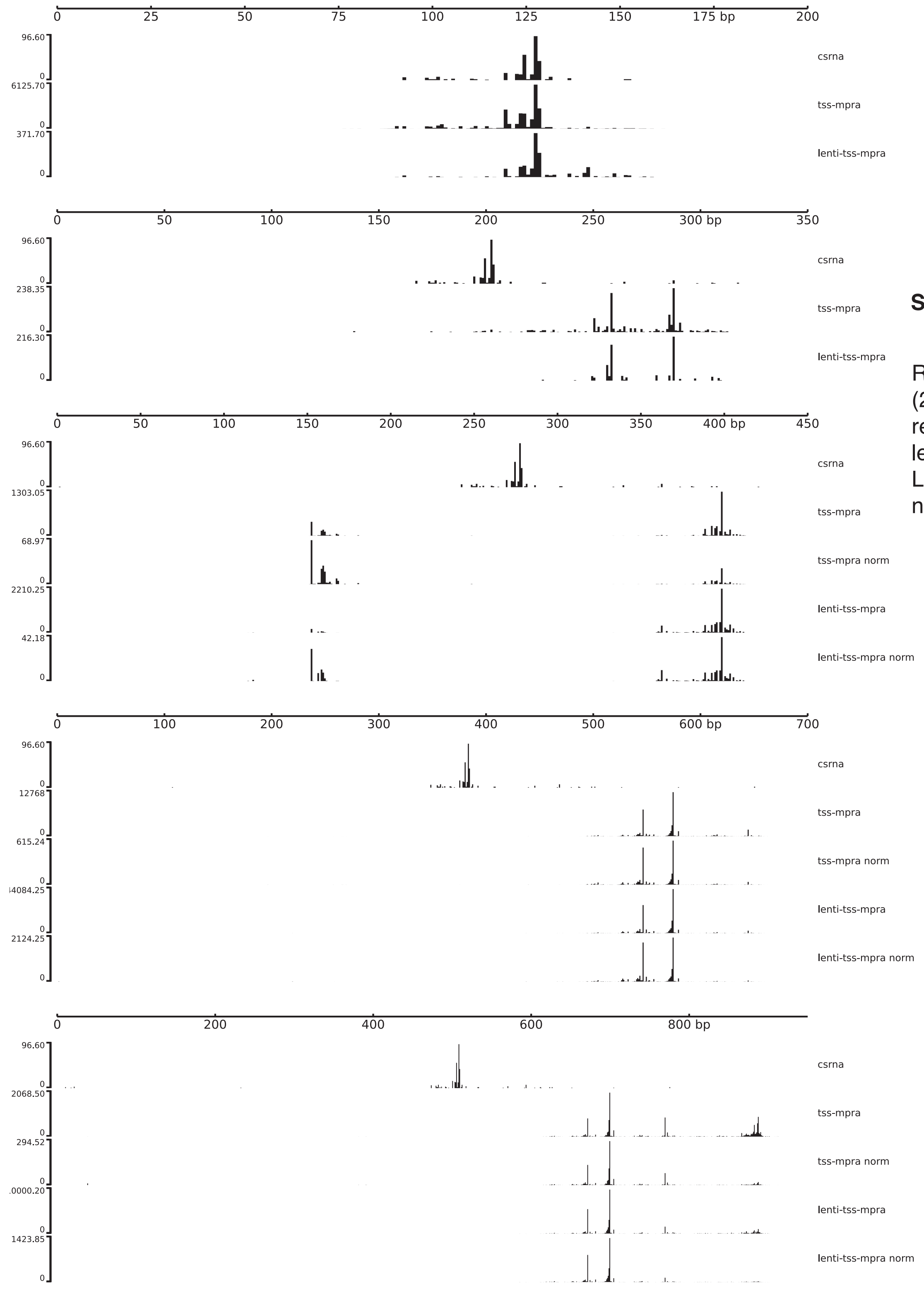

Supplementary Figure S13

Sequence 4

chr13:28658987-28659137

Supplementary Figure S13. TSS profiles across four different insert lengths.

RNA read counts at each bp of an insert across four different insert lengths (200bp, 350bp, 450bp, 700bp, and 950bp). The top track in each graph represents the endogenous TSS profile (csRNA-seq) while the tss-mpra and lenti-tss-mpra labeled tracks represent the TSS profiles from TSS-MPRA and Lenti-TSS-MPRA experiments respectively. Tracks labeled with ‘norm’ have been normalized for sequencing size bias.

Supplementary Figure S14

Sequence 5

chr15:43370948-43371098

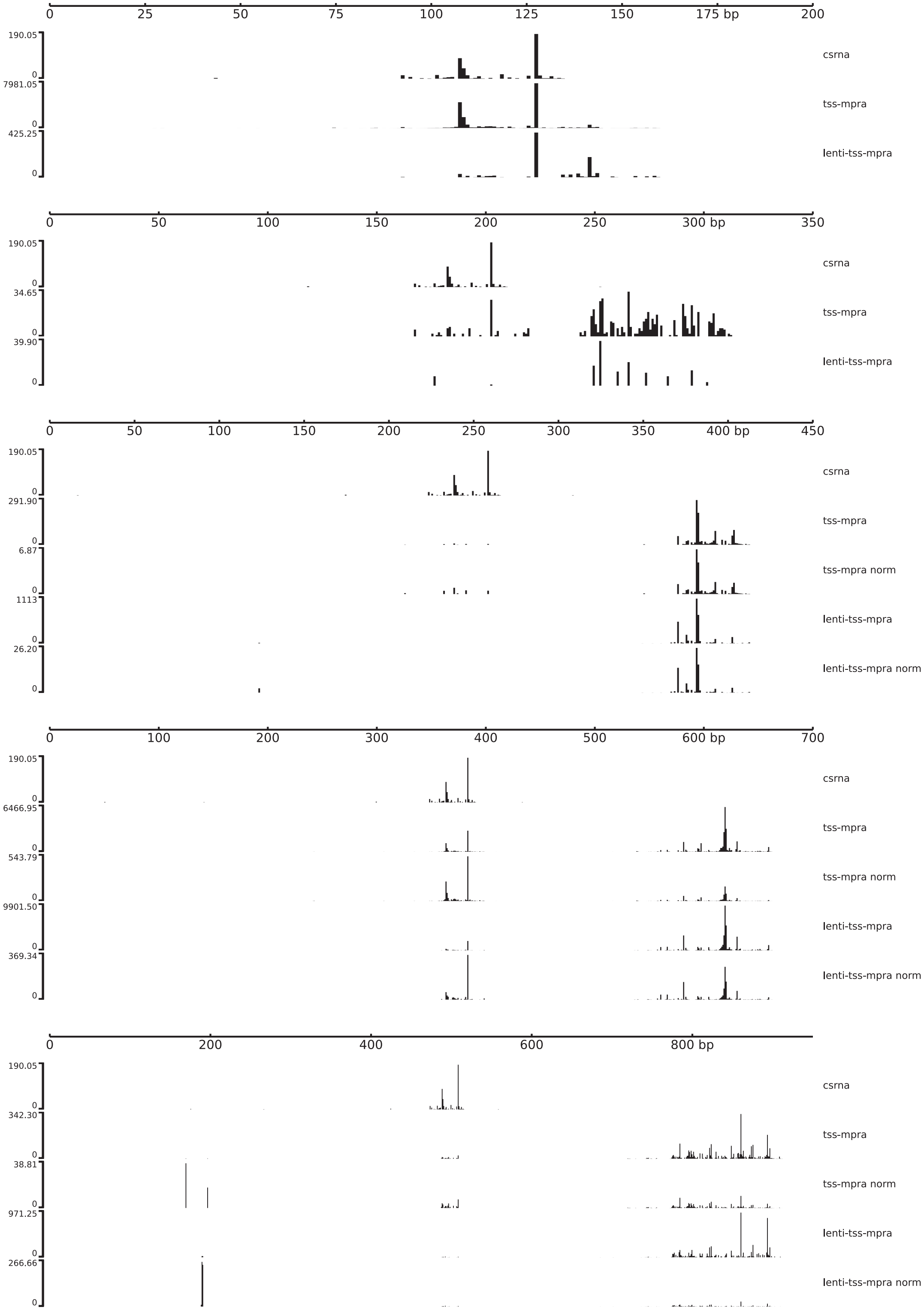

Supplementary Figure S14. TSS profiles across four different insert lengths.

RNA read counts at each bp of an insert across four different insert lengths (200bp, 350bp, 450bp, 700bp, and 950bp). The top track in each graph represents the endogenous TSS profile (csRNA-seq) while the tss-mpra and lenti-tss-mpra labeled tracks represent the TSS profiles from TSS-MPRA and Lenti-TSS-MPRA experiments respectively. Tracks labeled with ‘norm’ have been normalized for sequencing size bias.

Supplementary Figure S15

Sequence 6

chr5:119071034-119071184

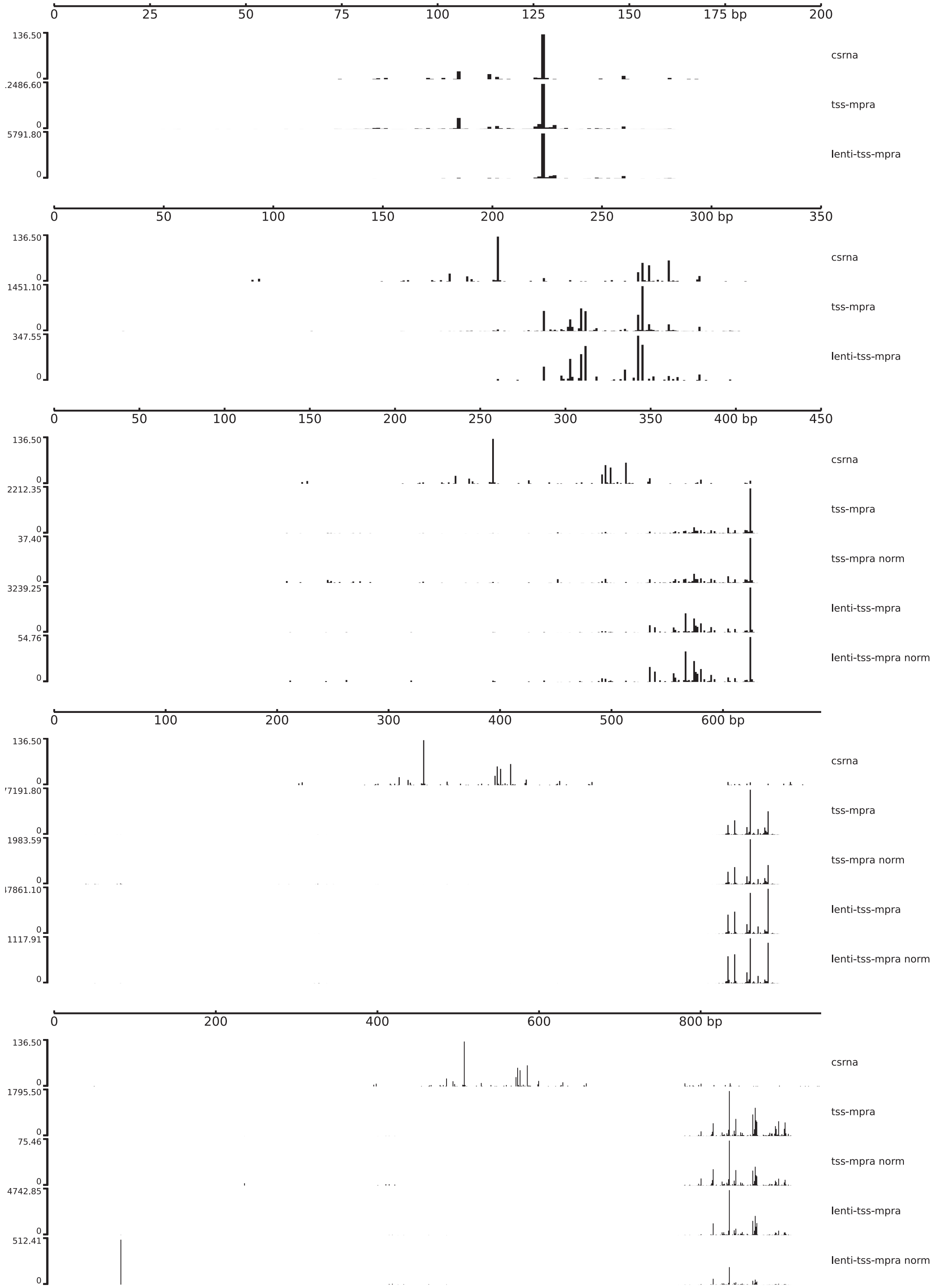

Supplementary Figure S15. TSS profiles across four different insert lengths.

RNA read counts at each bp of an insert across four different insert lengths (200bp, 350bp, 450bp, 700bp, and 950bp). The top track in each graph represents the endogenous TSS profile (csRNA-seq) while the tss-mpra and lenti-tss-mpra labeled tracks represent the TSS profiles from TSS-MPRA and Lenti-TSS-MPRA experiments respectively. Tracks labeled with ‘norm’ have been normalized for sequencing size bias.

Supplementary Figure S16

Sequence 7

chr5:149320819-149320969

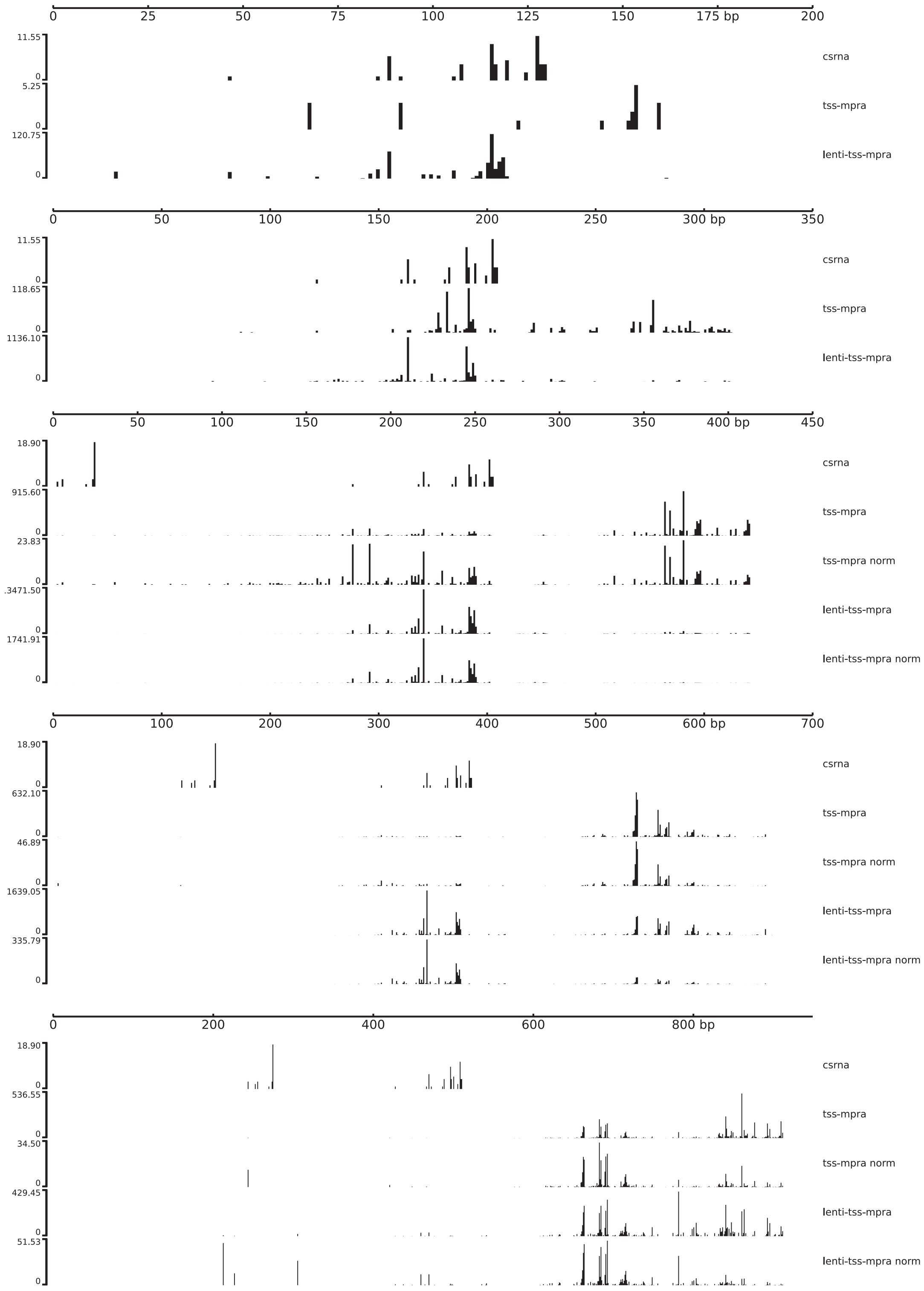

Supplementary Figure S16. TSS profiles across four different insert lengths.

RNA read counts at each bp of an insert across four different insert lengths (200bp, 350bp, 450bp, 700bp, and 950bp). The top track in each graph represents the endogenous TSS profile (csRNA-seq) while the tss-mpra and lenti-tss-mpra labeled tracks represent the TSS profiles from TSS-MPRA and Lenti-TSS-MPRA experiments respectively. Tracks labeled with ‘norm’ have been normalized for sequencing size bias.

Supplementary Figure S17

Sequence 8

chr6:131628156-131628306

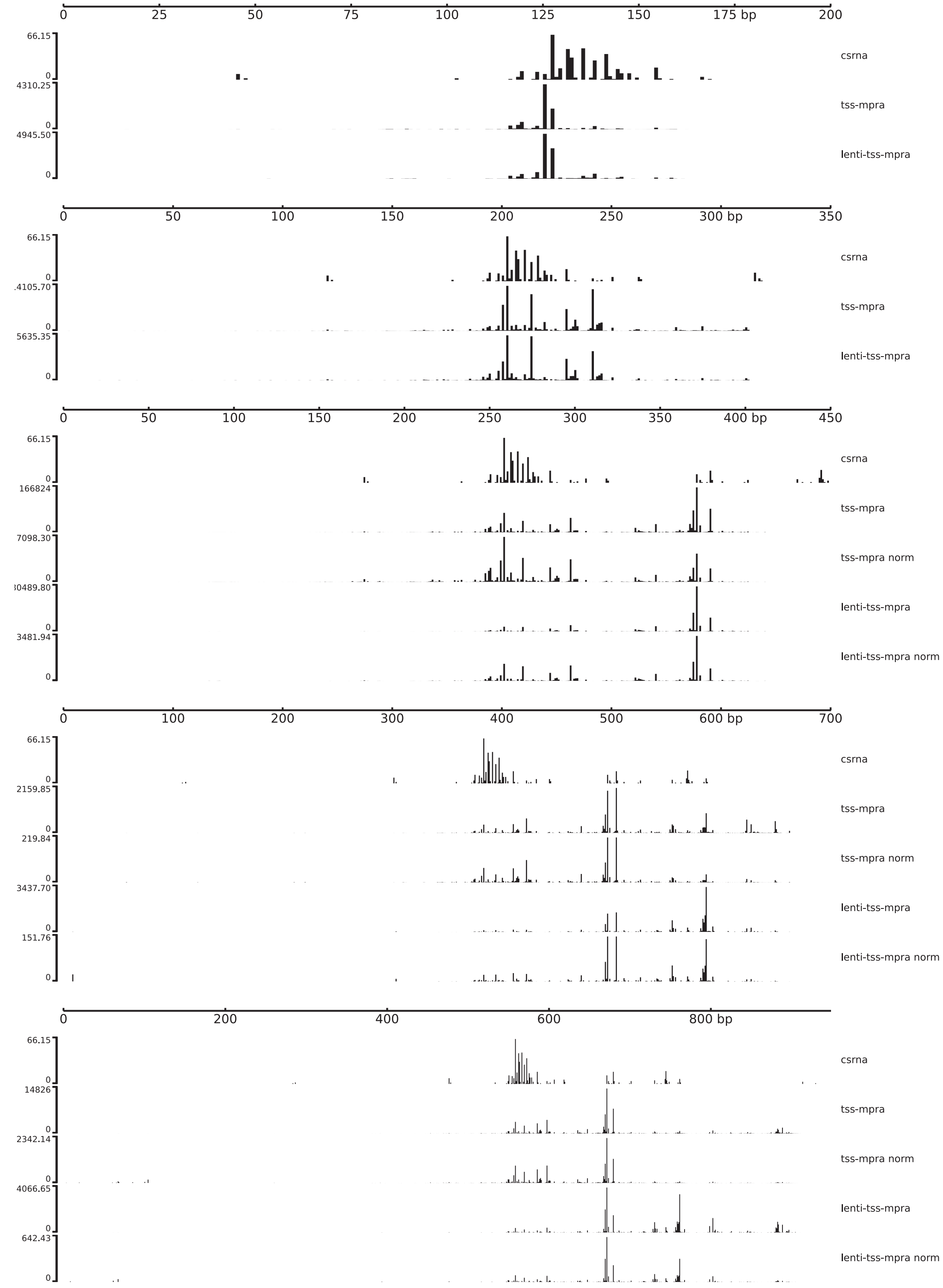

Supplementary Figure S17. TSS profiles across four different insert lengths.

RNA read counts at each bp of an insert across four different insert lengths (200bp, 350bp, 450bp, 700bp, and 950bp). The top track in each graph represents the endogenous TSS profile (csRNA-seq) while the tss-mpra and lenti-tss-mpra labeled tracks represent the TSS profiles from TSS-MPRA and Lenti-TSS-MPRA experiments respectively. Tracks labeled with ‘norm’ have been normalized for sequencing size bias.

Supplementary Figure S18

Sequence 9

chr3:126475816-126475966

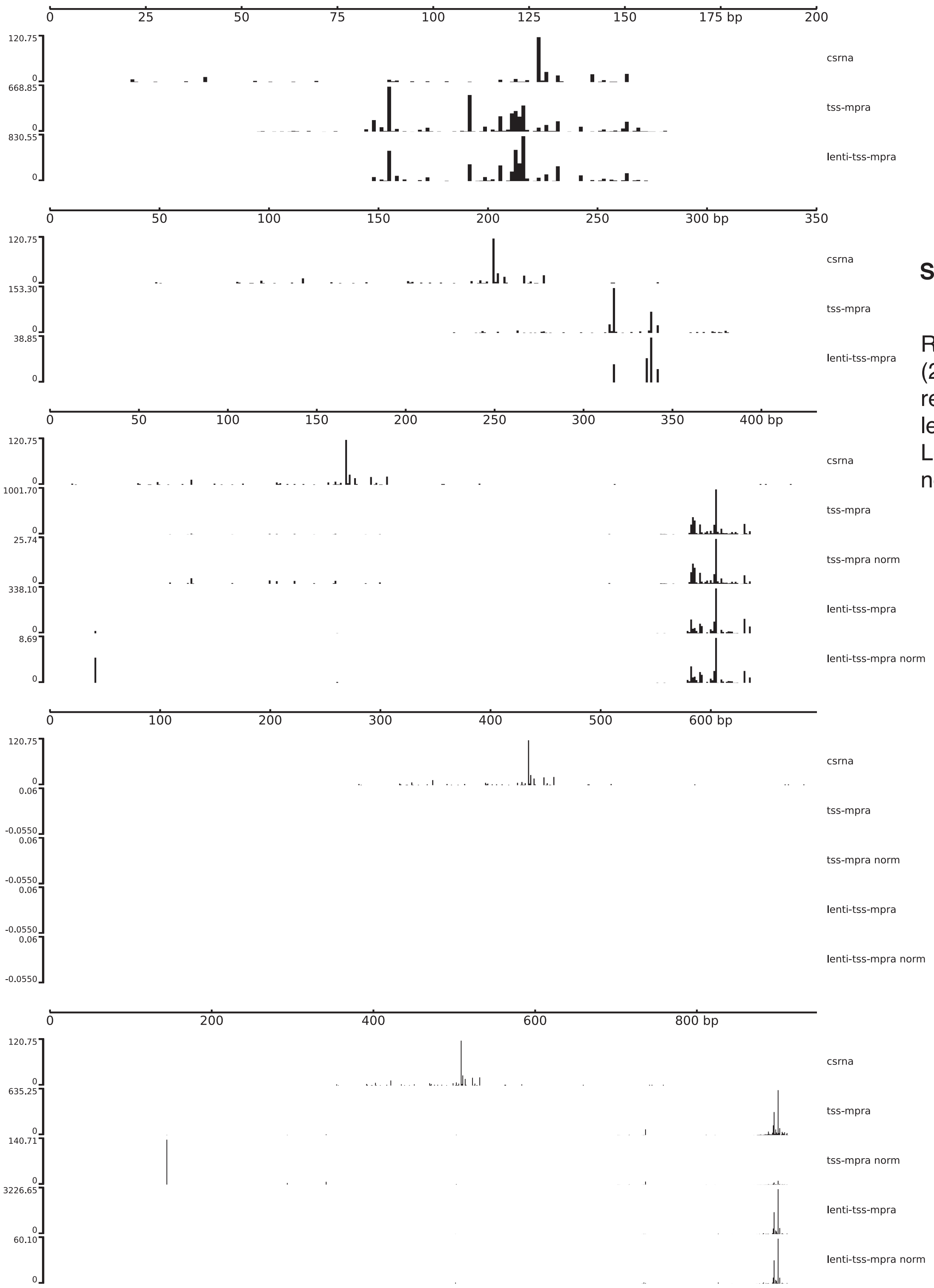

Supplementary Figure S18. TSS profiles across four different insert lengths.

RNA read counts at each bp of an insert across four different insert lengths (200bp, 350bp, 450bp, 700bp, and 950bp). The top track in each graph represents the endogenous TSS profile (csRNA-seq) while the tss-mpra and lenti-tss-mpra labeled tracks represent the TSS profiles from TSS-MPRA and Lenti-TSS-MPRA experiments respectively. Tracks labeled with 'norm' have been normalized for sequencing size bias.

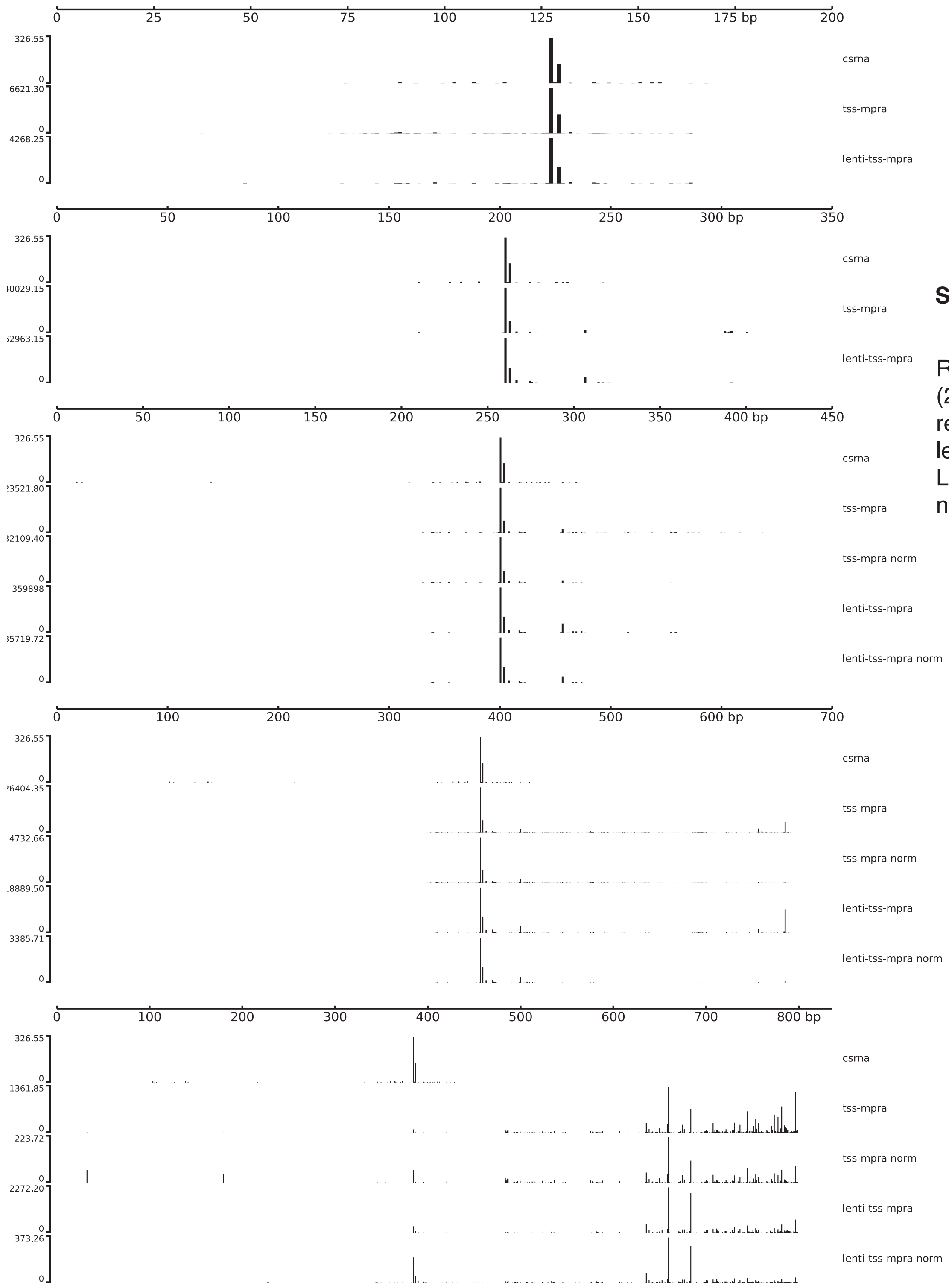

Supplementary Figure S19

Sequence 10

chr19:5718804-5718954

Supplementary Figure S19. TSS profiles across four different insert lengths.

RNA read counts at each bp of an insert across four different insert lengths (200bp, 350bp, 450bp, 700bp, and 950bp). The top track in each graph represents the endogenous TSS profile (csRNA-seq) while the tss-mpra and lenti-tss-mpra labeled tracks represent the TSS profiles from TSS-MPRA and Lenti-TSS-MPRA experiments respectively. Tracks labeled with ‘norm’ have been normalized for sequencing size bias.

Supplementary Figure S20

Sequence 11

chr2:231708455-231708605

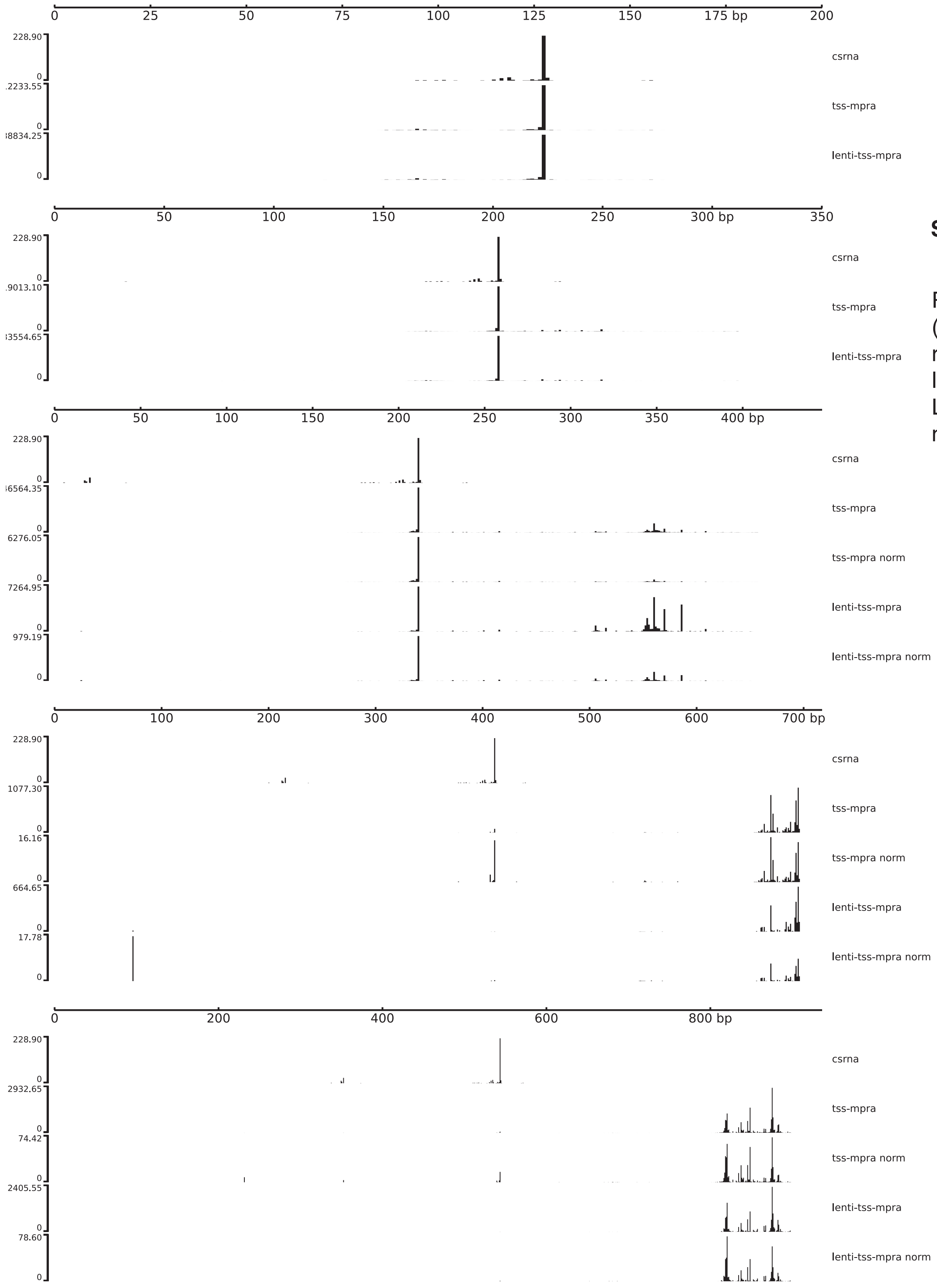

Supplementary Figure S20. TSS profiles across four different insert lengths.

RNA read counts at each bp of an insert across four different insert lengths (200bp, 350bp, 450bp, 700bp, and 950bp). The top track in each graph represents the endogenous TSS profile (csRNA-seq) while the tss-mpra and lenti-tss-mpra labeled tracks represent the TSS profiles from TSS-MPRA and Lenti-TSS-MPRA experiments respectively. Tracks labeled with ‘norm’ have been normalized for sequencing size bias.

Supplementary Figure S21

Sequence 12

chr6:26596710-26596860

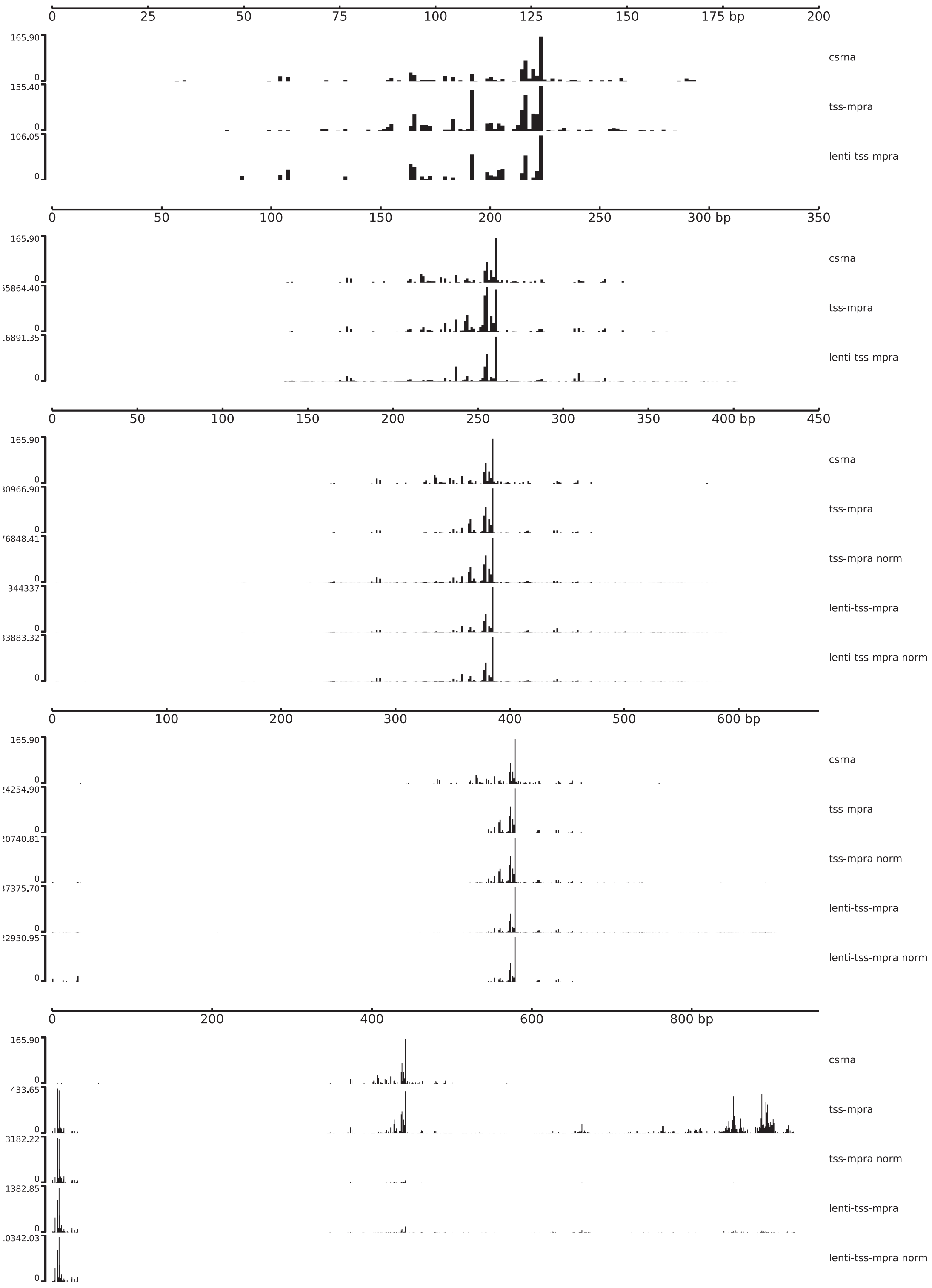

Supplementary Figure S21. TSS profiles across four different insert lengths.

RNA read counts at each bp of an insert across four different insert lengths (200bp, 350bp, 450bp, 700bp, and 950bp). The top track in each graph represents the endogenous TSS profile (csRNA-seq) while the tss-mpra and lenti-tss-mpra labeled tracks represent the TSS profiles from TSS-MPRA and Lenti-TSS-MPRA experiments respectively. Tracks labeled with 'norm' have been normalized for sequencing size bias.
