# Supplementary material for: Combining TSS-MPRA and sensitive TSS profile dissimilarity scoring to study the sequence determinants of transcription initiation
Source: Nucleic Acids Res. 2023 Jul 5;51(15):e80. doi: 10.1093/nar/gkad562 (PMC10450201; doi:10.1093/nar/gkad562)
Supplement: gkad562_Supplemental_Files [file gkad562_supplemental_files.zip › Supplementary_Table_Legends.docx]

**Supplementary Table S1. Insert sequence data for the short oligonucleotide pool used to generate the *epi-short* and *lenti-short* plasmid libraries.**

**Supplementary Table S2. Insert sequence data for the short oligonucleotide pool used to generate the *epi-long* and *lenti-long* plasmid libraries.**

**Supplementary Table S3. Forward and Reverse primer sequences used to generate 450-, 700-, and 950-bp sequence inserts.**

**Supplementary Table S4. Locus and group characteristics for the twelve sequences selected to test increased insert length.**
